# The long-term effects of genomic selection: 2. Changes in allele frequencies of causal loci and new mutations

**DOI:** 10.1093/genetics/iyad141

**Published:** 2023-07-28

**Authors:** Yvonne C J Wientjes, Piter Bijma, Joost van den Heuvel, Bas J Zwaan, Zulma G Vitezica, Mario P L Calus

**Affiliations:** Animal Breeding and Genomics, Wageningen University & Research, 6700 AH Wageningen, The Netherlands; Animal Breeding and Genomics, Wageningen University & Research, 6700 AH Wageningen, The Netherlands; Laboratory of Genetics, Wageningen University & Research, 6700 AH Wageningen, The Netherlands; Laboratory of Genetics, Wageningen University & Research, 6700 AH Wageningen, The Netherlands; UMR 1388 GenPhySE, INRAE, 31326 Castanet-Tolosan, France; Animal Breeding and Genomics, Wageningen University & Research, 6700 AH Wageningen, The Netherlands

**Keywords:** selection, genomic changes, allelic architecture, mutations, allele fixation, nonadditive effects, genetic variance, genomic selection, genomic prediction, GenPred, shared data resources

## Abstract

Genetic selection has been applied for many generations in animal, plant, and experimental populations. Selection changes the allelic architecture of traits to create genetic gain. It remains unknown whether the changes in allelic architecture are different for the recently introduced technique of genomic selection compared to traditional selection methods and whether they depend on the genetic architectures of traits. Here, we investigate the allele frequency changes of old and new causal loci under 50 generations of phenotypic, pedigree, and genomic selection, for a trait controlled by either additive, additive and dominance, or additive, dominance, and epistatic effects. Genomic selection resulted in slightly larger and faster changes in allele frequencies of causal loci than pedigree selection. For each locus, allele frequency change per generation was not only influenced by its statistical additive effect but also to a large extent by the linkage phase with other loci and its allele frequency. Selection fixed a large number of loci, and 5 times more unfavorable alleles became fixed with genomic and pedigree selection than with phenotypic selection. For pedigree selection, this was mainly a result of increased genetic drift, while genetic hitchhiking had a larger effect on genomic selection. When epistasis was present, the average allele frequency change was smaller (∼15% lower), and a lower number of loci became fixed for all selection methods. We conclude that for long-term genetic improvement using genomic selection, it is important to consider hitchhiking and to limit the loss of favorable alleles.

## Teaser

Genomic selection has revolutionized genetic improvement in animals and plants. Here, Wientjes *et al*. investigate the long-term effects of genomic selection on allele frequency changes of causal loci using stochastic simulations. Genomic selection rapidly changed allele frequencies, and the allele frequency change per locus was highly influenced by its effect and linkage with other loci. Moreover, selection fixed many favorable and unfavorable loci. For long-term genetic improvement using genomic selection, minimizing the loss of favorable alleles is important.

## Introduction

Genetic selection has been applied for many generations in animal and plant populations as well as in experimental species (e.g. mice, fruit flies, and butterflies). This has resulted in a considerable improvement in desirable traits of those populations (Beniwal *et al*. 1992; Dudley and Lambert 2003; Havenstein *et al*. 2003a, 2003b; Weber 1996). The aim of selection is to select the genetically most suitable individuals to produce the next generation. This will increase the frequency of alleles with a positive average effect on the desirable traits, which is known as genetic gain (Falconer and Mackay 1996; Walsh and Lynch 2018).

Selection has traditionally been based on pedigree and phenotypic information of the individuals and their relatives. In the last decade, pedigree selection has more and more been replaced by genomic selection (Hayes *et al*. 2009; Knol *et al*. 2016; Meuwissen *et al*. 2001, 2016; Wolc *et al*. 2016). The use of genomic selection has likely accelerated the changes in allele frequencies across generations in certain regions on the genome (Doekes *et al*. 2018; Heidaritabar *et al*. 2014; Liu *et al*. 2014). It has been hypothesized that genomic selection mainly focuses on genes with a large contribution to the genetic variance [i.e. genes with a large effect and high minor allele frequency (MAF)] and tends to ignore the regions with a smaller contribution to the genetic variance of the trait of interest (Bijma 2012; Goddard 2009). As a consequence, the risk of losing rare favorable alleles has increased since the introduction of genomic selection (De Beukelaer *et al*. 2017; Jannink 2010; Liu *et al*. 2014). Monitoring and understanding the impact of selection on the changes in allele frequency of loci are important for investigating the long-term effects of selection. Since the vast majority of causal loci are unknown, simulation studies can contribute to better understand and quantify the changes at causal loci and how these are affected by selection approaches.

In a previous study (Wientjes *et al*. 2022), we have used simulations to investigate the long-term effects of selection on genetic gain, genetic variance, and genetic architecture. The main conclusion was that the short-term response was highest for genomic selection. The long-term response to selection was larger with phenotypic selection than with genomic selection, because genomic selection lost more genetic variance. Genomic selection always outperformed pedigree selection and lost a similar amount of genetic variance. Both genetic gain and loss of genetic variance depended on the presence of nonadditive effects, such as dominance and epistasis. When epistasis was present, selection substantially changed the statistical additive effects of the trait over time. Therefore, epistasis can change the pressure and direction of selection on an allele over time, which resulted in a lower average allele frequency change. However, we did not study the pattern of allele frequency change in detail in our previous study.

Although epistatic interactions between causal loci are known to be common (Carlborg and Haley 2004; Carlborg *et al*. 2006; Flint and Mackay 2009; Huang *et al*. 2012), not much is known about the complete genetic interaction network in animals and plants. More elaborate information is available for the genetic interaction network in yeast (Boone *et al*. 2007; Costanzo *et al*. 2016; Tong *et al*. 2004), where epistatic interactions were described between 90% of the identified causal loci. Most of the loci were involved in only a few interactions, while some loci were involved in many interactions. A similar pattern, although studied in less detail, is described for other laboratory species, such as *Caenorhabditis elegans* (Lehner *et al*. 2006), *Drosophila* (Huang *et al*. 2012), and mice (Tyler *et al*. 2017). Moreover, protein–protein binding interaction networks are similar in yeast, animals, including humans, and plants. Therefore, Boone *et al*. (2007) and Mackay (2014) argued that it is likely that the observed genetic interaction network in yeast may reflect such interaction networks in other species as well.

When investigating the long-term effects of selection, it is important to also consider new mutations. This is because after sufficient time, the response to selection will be completely driven by the variation due to new mutations (Walsh and Lynch 2018), and without mutations, the genetic variance will rapidly deplete. The potential of selection methods to maintain and utilize the variance generated by new mutations after 20 generations of selection differs between pedigree and genomic selection methods and is especially limited when no own performance (OP) information is used in the selection criterion (Mulder *et al*. 2019). However, little is known about the potential of selection methods to maintain and exploit new mutations over longer time.

To date, the long-term effects of the selection method and nonadditive effects (dominance and epistasis) on the change in allelic architecture of complex traits under selection are still unknown. More information about those changes is essential to better understand and predict the long-term impact of selection. Therefore, the aim of this study is to compare different selection methods with respect to changes in allelic architecture of complex traits. To this end, we simulated 50 generations of phenotypic, pedigree, and genomic selection for three genetic architectures with only additive, additive and dominance, or additive, dominance, and epistatic effects and analyzed the allelic architecture through the changes in allele frequencies of causal loci and new mutations.

## Materials and methods

We used the simulated data from our previous study (for details see Wientjes *et al*. 2022). In brief, a historical population was simulated in QMSim software (Sargolzaei and Schenkel 2009). From this historical population, a number of individuals were selected to form the starting population under selection, which was further simulated using our own developed Fortran program. The simulated data represent a livestock population in terms of allele frequency distribution, which was strongly U-shaped for segregating loci as typically observed in sequence data (Bolormaa *et al*. 2019; Daetwyler *et al*. 2014; Eynard *et al*. 2015; Heidaritabar *et al*. 2016). Moreover, the linkage disequilibrium (LD) pattern was comparable to livestock populations, with reasonably strong linkage at short distances on the genome (Andreescu *et al*. 2007; Badke *et al*. 2012; Veroneze *et al*. 2013; Supplementary Figs. 1.1 and 1.2 in Supplementary File 1). In the first 50 generations after the historical population, the best 100 females and 100 males were randomly mated using a mating ratio of 1:1 and a litter size of 10 (5 females and 5 males). Thereafter, 15 scenarios were simulated that contained all combinations of 3 genetic models and 5 selection methods that were run for another 50 generations, and the results of those sets of simulations are presented in this study. All scenarios were replicated 20 times.

### Genome and mutations

The genome of the simulated population contained 10 chromosomes of 100 cm each, with on average one recombination event per offspring chromosome. In the last historical generation, a marker set of 20,000 segregating loci was selected with a uniform allele frequency distribution (see Supplementary Fig. 1.3 in Supplementary File 1 for the allele frequency distribution of markers before and after selection). Given that roughly 1/3 of a livestock genome was simulated, the chosen marker density was similar to a commonly used 60k chip in livestock. A set of 2,000 segregating causal loci was randomly selected with a U-shaped allele frequency distribution. Moreover, a set of 4,000 nonsegregating loci (i.e. that showed no variation at the start of selection) was randomly selected to serve as locations for causal mutations. Each individual had on average 0.6 new mutations that were causal to the simulated trait, sampled from a Poisson distribution. This resulted in a mutational variance of ∼0.001$\sigma_{e}^{2}$ when assuming only additive effects (as explained later), which agrees with estimates from experimental populations (Hill 1982a; Houle *et al*. 1996; Lynch and Walsh 1998). For computational reasons, the loci and effects for new mutations were recycled while treating every time the mutation as a new mutation. Thus, for each new mutation, a locus that did not segregate at that moment was selected from all 4,000 potential locations for causal mutations while maximizing the time between 2 mutations at the same locus. This resulted in using each locus on average once in every 6–7 generations as a new mutation. We believe that the recycling of loci for mutations did not impact the results of our study, because the number of potential locations was reasonably large compared to the number of mutations generated every generation, and the majority of mutations (80%) were already lost in the first generation as a result of drift and not related to the effect of the mutation (Supplementary Table 2.1 in Supplementary File 2).

### Complex traits

For all causal loci (including loci for mutations), functional effects were assigned using one of 3 genetic models, namely a model with only additive effects (A), a model with additive and dominance effects (AD), and a model with additive, dominance, and epistatic effects (ADE). Additive and dominance effects were simulated for all causal loci, based on established approaches (i.e. Duenk *et al*. 2020; Wellmann and Bennewitz 2011). Additive effects (*a*) were sampled from *N*(0,1). Dominance effects (*d*) were simulated to be proportional to the additive effects by first sampling a dominance degree (dd) from *N*(0.2,0.3) and subsequently using $d_{i}=dd_{i}|a_{i}|$ for all loci *i*.

Only pairwise epistatic effects were simulated based on the epistatic network described in yeast, where 90% of the causal loci were involved in one or more interactions (Costanzo *et al*. 2016). In this network, most loci were involved in a few interactions, and a few loci were involved in many interactions. Epistatic effects (*e*) were independently simulated for the 9 possible genotype combinations of a pairwise interaction and scaled to be proportional to the additive effects of both loci. Thus, for the interaction between loci *i* and *j*, an epistatic degree (*ε*) was sampled from *N*(0,0.45) for each possible genotype combination, and the epistatic effect was calculated as $e=\varepsilon \sqrt{\left|a_{i}a_{j}\right|}$.

Based on the functional effects and the genotypes, a total genotypic value (TGV) was calculated for each individual. A residual, sampled from a normal distribution using a broad sense heritability of 0.4, was added to calculate the phenotypic value. The simulated functional additive, dominance, and epistatic effects were used to compute the statistical additive (*α*) and dominance (*δ*) effects for each causal locus based on the allele frequencies of the causal loci, using the natural and orthogonal interaction approach (NOIA) (Álvarez-Castro and Carlborg 2007; Vitezica *et al*. 2017). The statistical additive effect (also known as allele substitution effect) represents the average effect when an allele is substituted by the other allele (Falconer and Mackay 1996). In contrast to the functional effects, statistical additive effects can change across generations, because they depend on the allele frequencies in that generation (Falconer and Mackay 1996). The sum of statistical additive effects is equal to the total additive genetic value (i.e. breeding value) of an individual. We estimated for each individual the total additive genetic value across all loci *i* as $A=\sum w_{a_{i}}\alpha_{i}$, with the following:


$$w_{a_{i}}=\{\begin{array}{c} \;p_{Bb}+2p_{bb}\\ \;p_{Bb}+2p_{bb}-1\\ \;p_{Bb}+2p_{bb}-2 \end{array}\mathrm{for}\;\mathrm{genotypes}\{\begin{array}{c} BB\\ Bb\\ bb \end{array},$$

where *p_BB_*, *p_Bb_*, and *p_bb_* represent the frequencies of the genotypes *BB*, *Bb*, and *bb* for locus *B*. The additive genetic variance was the variance in *A* among individuals.

The statistical dominance effects were used to compute the total dominance deviation across all loci *i* of each individual as $D=\sum w_{d_{i}}\delta_{i}$, with the following:


$$w_{d_{i}}=\{\begin{array}{c} \frac{-2p_{Bb}p_{bb}}{\;p_{BB}+p_{bb}-{\left(\;p_{BB}-p_{bb}\right)}^{2}}\\ \frac{4p_{BB}p_{bb}}{\;p_{BB}+p_{bb}-{\left(\;p_{BB}-p_{bb}\right)}^{2}}\\ \frac{-2p_{BB}p_{Bb}}{\;p_{BB}+p_{bb}-{\left(\;p_{BB}-p_{bb}\right)}^{2}} \end{array}\mathrm{for}\;\mathrm{genotypes}\{\begin{array}{c} BB\\ Bb\\ bb \end{array}.$$

The dominance genetic variance was the variance in *D* among individuals. The total genetic variance minus the additive and dominance variance is the epistatic variance. For genetic model ADE, almost 50% of the variation at the functional level was a result of the epistatic effects; however, at the statistical level, more than 60% of the variance was additive and only 5% epistatic.

### Selection process

We used 5 artificial selection methods. The first method, RANDOM, selected in each generation randomly the parents of the next generation. The second method, MASS, selected the parents based on their own phenotypes. The other 3 methods selected parents based on estimated breeding values using best linear unbiased prediction (BLUP). With PBLUP_OP, breeding values were estimated using pedigree information from the last 8 generations and phenotypes from the last 3 generations, including own performance (OP) of the selection candidates. With GBLUP_NoOP and GBLUP_OP, breeding values were estimated using marker genotypes and phenotypes from the last 3 generations, either excluding (NoOP) or including (OP) the OP of the selection candidates. Breeding values were estimated with the MTG2 software (Lee and van der Werf 2016), simultaneously with the variance components. Since commercial models for genetic improvement of animals and plants are mainly based on additive models (Crossa *et al*. 2010; Goddard *et al*. 2010), our breeding value estimation model included a fixed mean, random additive genetic effects, random litter effects, and residuals. The random litter effect was included to prevent that the resemblance between full sibs due to nonadditive genetic effects created bias in the estimated variance components and breeding values.

### Change in allelic architecture

We investigated the changes in allelic architecture of causal loci underlying a complex trait over 50 generations of drift and selection. First, we investigated the general pattern of change in allele frequency of causal loci across the genome and the relation between allele frequency change and statistical additive effect. The statistical additive effect (*α*) is the *partial* regression coefficient of the TGV on allele count [$z_{ik}=(0,1,\mathrm{or}\;2)$ for individual *k*], $TGV=\mathrm{intercept}+z_{i}\alpha_{i}+\sum_{\;j\neq i}\;z_{j}\alpha_{j}+e$, in which the effect of linked loci is captured by the other loci $\left(\sum_{\;j\neq i}\;z_{j}\alpha_{j}\right)$ and therefore not included in the estimated effect of the locus of interest $\left(\alpha_{i}\right)$. However, it is well known that linked loci can have a strong impact on the selection pressure on a locus and thereby also on allele frequency change and probability of fixation (Walsh and Lynch 2018). Therefore, we also investigated the relationship between allele frequency change and the *apparent* effect of a causal locus, which also included the effects of linked loci. Apparent effects were estimated in each generation for each locus as the *simple* regression coefficient of the total additive genetic value (i.e. breeding value) on the gene content at the locus, $A=\mathrm{intercept}+z_{i}\alpha_{\mathrm{Apparent},i}+e$, where ***A*** is a vector with the total additive genetic values of all individuals in 1 generation, $z_{i}$ is a vector with the allele count of locus *i* of all individuals, $\alpha_{\mathrm{Apparent},i}$ is the apparent effect of locus *i*, and ***e*** is a vector with residuals.

Then, we zoomed in on the causal loci with either a large (>0.9) or small (<0.01) change in allele frequency across the 50 generations of selection and investigated the characteristics of those loci, such as average functional and statistical effects, interaction network, and allele frequency pattern. The cutoff values for the change in allele frequencies were chosen such that a reasonable number of loci met the criteria (see Supplementary Fig. 1.4 in Supplementary File 1 for the distribution in allele frequency change). The number of loci with an allele frequency change < 0.01 was very large, because loci initially close to fixation could not change in allele frequency after becoming fixed. Therefore, we used an additional criterion that the starting MAF had to be at least 0.05 for the loci with a small allele frequency change.

Thereafter, we investigated the characteristics of the causal loci that became fixed or lost during selection while using the criterion that the minimal change in allele frequency had to be 0.2 to disregard loci that were already close to fixation or loss in the initial generation. In addition, we looked at the probability of loci to become fixed or lost over 50 generations of selection as a function of their starting allele frequency and compared the pattern with the expected pattern under drift. The expected pattern under drift was obtained from additional simulations with no selection and the same effective population size (*N*_e_). The *N*_e_ for each selection scenario was estimated from the pedigree kinship coefficient, using $N_{e}=\frac{1}{2\Delta f}$ (Caballero 1994), where Δf is the rate of kinship from generations 0–50 based on the off-diagonal elements of the pedigree relationship matrix containing all 50 generations. Then new simulations were run with random selection and the same *N*_e_ as in the selection scenarios, by adjusting the number of parents (Supplementary Table 3.1 and text in Supplementary File 3).

Finally, we investigated the number of causal mutations that appeared between generations 0 and 49, and still segregated in generation 50, as well as their allele frequency in generation 50.

## Results

### General characteristics of selection

For ease of understanding the results, Table 1 shows the change in allele frequency of causal loci and phenotypic value, and the proportion of genetic variance lost over 50 generations for all 15 scenarios, with more details in Wientjes *et al*. (2022). When only additive (model A) or additive and dominance (model AD) effects were present in the population, the overall phenotypic change over 50 generations of selection was highest for GBLUP_OP, followed by MASS, PBLUP_OP, and GBLUP_NoOP (Table 1). When also epistatic effects were present (model ADE), MASS selection outperformed GBLUP_OP after ∼45 generations. As expected, RANDOM selection did not change the average phenotype over the 50 generations, and the short-term response to selection was always lowest for MASS. The genetic variance was constant under RANDOM selection, while ∼60% of the genetic variance was lost with MASS selection. The other selection methods resulted in losing 70–85% of the genetic variance and lost slightly more genetic variance when only additive effects were present.

**Table 1.** Change in average phenotype, genetic variance, and allele frequency across 50 generations of selection for the 5 selection methods and 3 genetic models*^a^*.

	Phenotypic change*^b^*	Proportion of genetic variance lost	Average change in allele frequency
Model A			
RANDOM	0.02 (0.11)	0.03 (0.03)	0.023 (0.000)
MASS	27.31 (0.44)	0.60 (0.01)	0.054 (0.001)
PBLUP_OP	27.02 (0.46)	0.79 (0.01)	0.062 (0.000)
GBLUP_NoOP	25.25 (0.45)	0.82 (0.01)	0.063 (0.001)
GBLUP_OP	29.37 (0.42)	0.83 (0.00)	0.065 (0.001)
Model AD			
RANDOM	0.02 (0.12)	0.02 (0.03)	0.023 (0.000)
MASS	26.80 (0.37)	0.56 (0.02)	0.053 (0.000)
PBLUP_OP	25.43 (0.37)	0.74 (0.01)	0.061 (0.000)
GBLUP_NoOP	23.54 (0.34)	0.81 (0.01)	0.064 (0.000)
GBLUP_OP	27.68 (0.29)	0.81 (0.01)	0.065 (0.000)
Model ADE			
RANDOM	0.05 (0.11)	0.00 (0.03)	0.023 (0.000)
MASS	20.87 (0.27)	0.56 (0.02)	0.042 (0.000)
PBLUP_OP	18.76 (0.29)	0.74 (0.01)	0.053 (0.000)
GBLUP_NoOP	16.79 (0.21)	0.75 (0.01)	0.054 (0.000)
GBLUP_OP	20.21 (0.26)	0.76 (0.01)	0.055 (0.000)

The 5 selection methods were as follows: RANDOM selection, MASS selection, PBLUP selection with own performance (PBLUP_OP), GBLUP selection without own performance (GBLUP_NoOP) or with own performance (GBLUP_OP). The 3 genetic models were a model with only additive effects (A), with additive and dominance effects (AD), or with additive, dominance, and epistatic effects (ADE).

Results are shown as averages across replicates with standard errors across replicates between brackets.

Expressed in base-generation additive genetic standard deviations.

### Change in allelic architecture of causal loci

Here, we investigate the average change in allelic architecture of causal loci for the different scenarios and how allele frequency change was spread across the genome. With RANDOM selection, the average absolute change in allele frequency across all loci was 0.023, which represents the effect of drift (Table 1). As expected, selection resulted in a larger change in allele frequency. Under genetic models A and AD, the change in allele frequency was ∼2.3 times higher with MASS, ∼2.7 times higher with PBLUP_OP, ∼2.8 times higher with GBLUP_NoOP, and ∼2.9 times higher with GBLUP_OP than the change due to drift alone. The presence of epistasis resulted in a ∼15% lower change in allele frequencies. Across the 50 generations, MASS had the lowest average change in allele frequency and among the highest cumulative genetic gains (Table 1); therefore, MASS showed the smallest change in allele frequency per unit of genetic gain.

For RANDOM, allele frequencies of causal loci only changed due to drift, which resulted in a balanced allele frequency change across the genome with most allele frequency changes in the range of 0–0.25, with only very few changes above 0.4 (Supplementary Fig. 1.5 in Supplementary File 1). With selection, larger allele frequency changes up to 1 were observed, indicating that some (0.02% for PBLUP_OP, GBLUP_NoOP, and GBLUP_OP; 0.00% for MASS) new causal mutations became fixed within 50 generations. The vast majority (∼80%) of allele frequency changes were, however, still in the range of 0–0.25 with selection. Selection also resulted in allele frequency changes at markers, which we could track for the genomic selection scenarios (Supplementary Fig. 1.6 in Supplementary File 1). As expected, some peaks became visible where a causal locus and its surrounding markers together changed substantially in allele frequency.

As expected, the average change in allele frequency was larger for causal loci with a larger initial heterozygosity, and this relationship became stronger when selection was more accurate (Supplementary Fig. 1.7 in Supplementary File 1). With random selection, the maximum change in allele frequency over 20 replicates was also larger for loci with a higher initial heterozygosity. With selection, however, the maximum achieved change in allele frequency was closer to the maximum possible change in allele frequency and, therefore, higher for loci with a lower initial expected heterozygosity. A more thorough investigation of the characteristics of loci with either a small (<0.01) or a large (>0.9) change in allele frequency is described in Supplementary File 4 (text; Supplementary Figs. 4.1 and 4.2 and Tables 4.1–4.3 in Supplementary File 4), which shows a much lower number of loci with a large allele frequency change (>0.9) for genetic model ADE compared to models A and AD.

### Allele frequency change vs effect size of causal loci

**Fig. 1.**
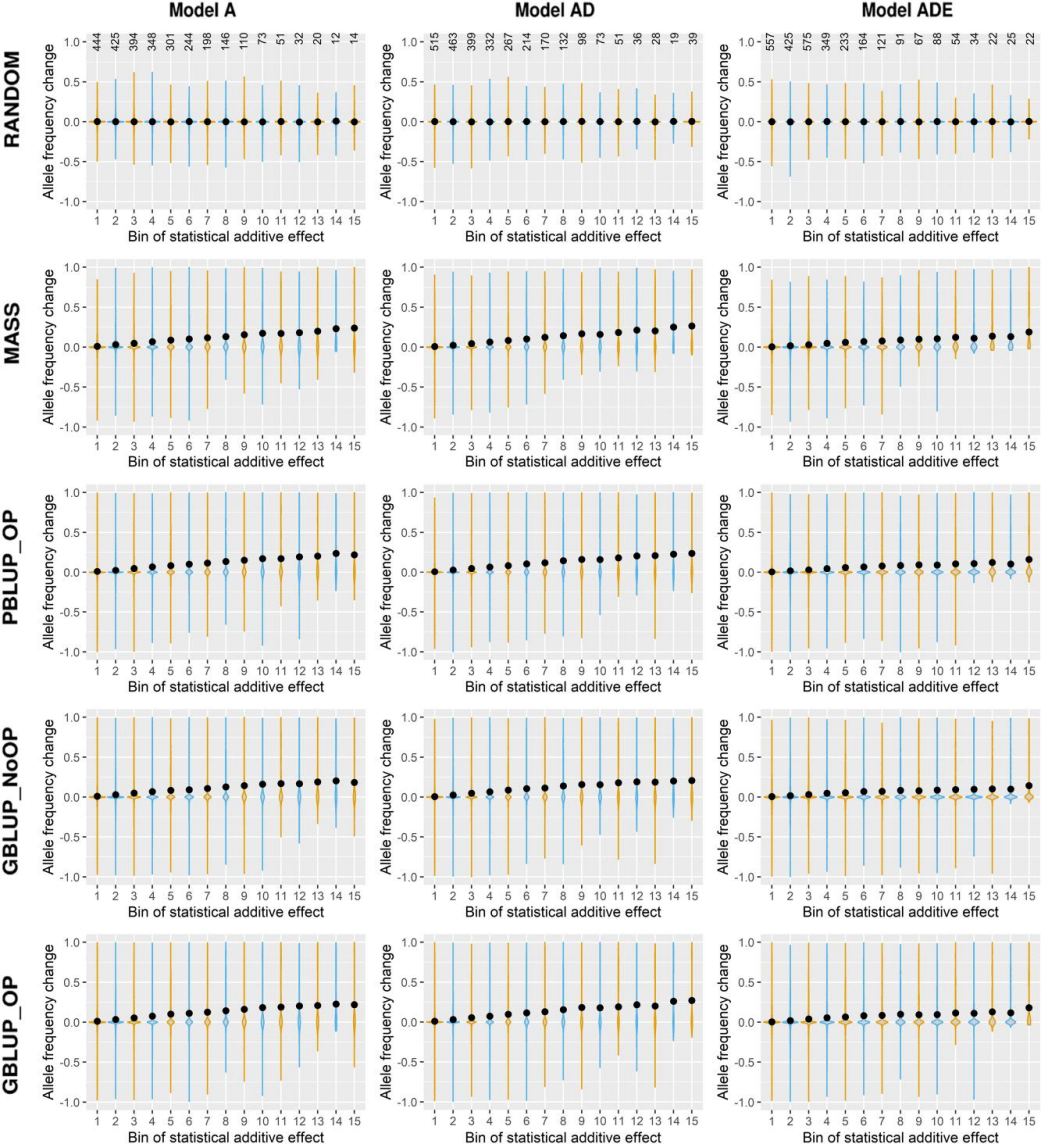
Change in allele frequency vs the size of the base-generation statistical additive effect (alpha), for the 5 selection methods and 3 genetic models. Loci are divided in bins based on their statistical additive effect in generation 0, where bin 1 contains the loci with the smallest statistical additive effect and bin 15 contains the loci with the largest statistical additive effect. The dots represent the average change in allele frequency for each of the bins. With only additive or additive and dominance effects, the following ranges in values of statistical additive effects were used: bin 1: <0.2; bin 2: 0.2–0.4; bin 3: 0.4–0.6; bin 4: 0.6–0.8; bin 5: 0.8–1.0; bin 6: 1.0–1.2; bin 7: 1.2–1.4; bin 8: 1.4–1.6; bin 9: 1.6–1.8; bin 10: 1.8–2.0; bin 11: 2.0–2.2; bin 12: 2.2–2.4; bin 13: 2.4–2.6; bin 14: 2.6–2.8; and bin 15: >2.8. With additive, dominance, and epistasis effects, the following ranges in values of statistical additive effects were used: bin 1: <0.5; bin 2: 0.5–1.0; bin 3: 1.0–2.0; bin 4: 2.0–3.0; bin 5: 3.0–4.0; bin 6: 4.0–5.0; bin 7: 5.0–6.0; bin 8: 6.0–7.0; bin 9: 7.0–8.0; bin 10: 8.0–10.0; bin 11: 10.0–12.0; bin 12: 12.0–14.0; bin 13: 14.0–16.0; bin 14: 16.0–20.0; and bin 15: >20.0. Positive allele frequency changes indicate an increase in frequency of the favorable allele, and negative allele frequency changes an increase in frequency of the unfavorable allele based on the allele substitution effects in generation 0. The 5 selection methods were as follows: RANDOM selection, MASS selection, PBLUP selection with own performance (PBLUP_OP), GBLUP selection without own performance (GBLUP_NoOP) or with own performance (GBLUP_OP). The 3 genetic models were a model with only additive effects (A), with additive and dominance effects (AD), or with additive, dominance, and epistatic effects (ADE). Results are given across all 20 replicates. The average number of loci per replicate in each bin is indicated at the top.

In this section, we investigate how the change in allele frequency of causal loci was related to the effect of the locus. Given that only drift changed the allele frequency in the RANDOM scenario, the average change in allele frequency, represented by the black dots in Fig. 1, was around 0 for all bins of causal loci based on their base-generation statistical additive effect. For the scenarios with selection, loci with a larger statistical additive effect showed on average a larger change in allele frequency for all genetic models. In genetic model A, the average initial allele frequency in each of the bins was similar, because additive effects were sampled independently from the allele frequency. Therefore, the correlation between statistical additive effect and allele frequency change also holds when accounting for the initial allele frequency in generation 0. Moreover, the maximum negative change in allele frequency (i.e. an increase in frequency of the unfavorable allele) was smaller for loci with a larger statistical additive effect. The presence of epistasis made the trend between allele frequency change and statistical additive effect less clear, as is also visible in the correlation coefficients between the change in allele frequency and the statistical additive effect in generation 0, which were lower for model ADE (Supplementary Table 2.2 in Supplementary File 2). This less clear trend is likely a result of changes in the statistical additive effect across generations which resulted in a large proportion of loci changing bin number across generations (24.7–43.0% of loci after 10 generations, and 37.9–58.2% after 50 generations; Supplementary Table 2.3 in Supplementary File 2) when epistasis was present.

We also quantified the trend between allele frequency change and initial allele substitution effect scaled by the statistical additive genetic standard deviation in generation 0. The regression coefficients of this trend ranged from 1.5 to 2.2 for genetic models A and AD and from 0.8 to 1.1 for genetic model ADE, while the correlation coefficient ranged from 0.33 to 0.42 for genetic models A and AD and from 0.24 to 0.33 for genetic model ADE (Supplementary Table 2.2 in Supplementary File 2). Surprisingly, however, the correlation between the allele frequency change from 1 generation to the next and the statistical additive effect was very low for all selection methods in all generations (<0.05; Supplementary Fig. 1.8 in Supplementary File 1).

**Fig. 2.**
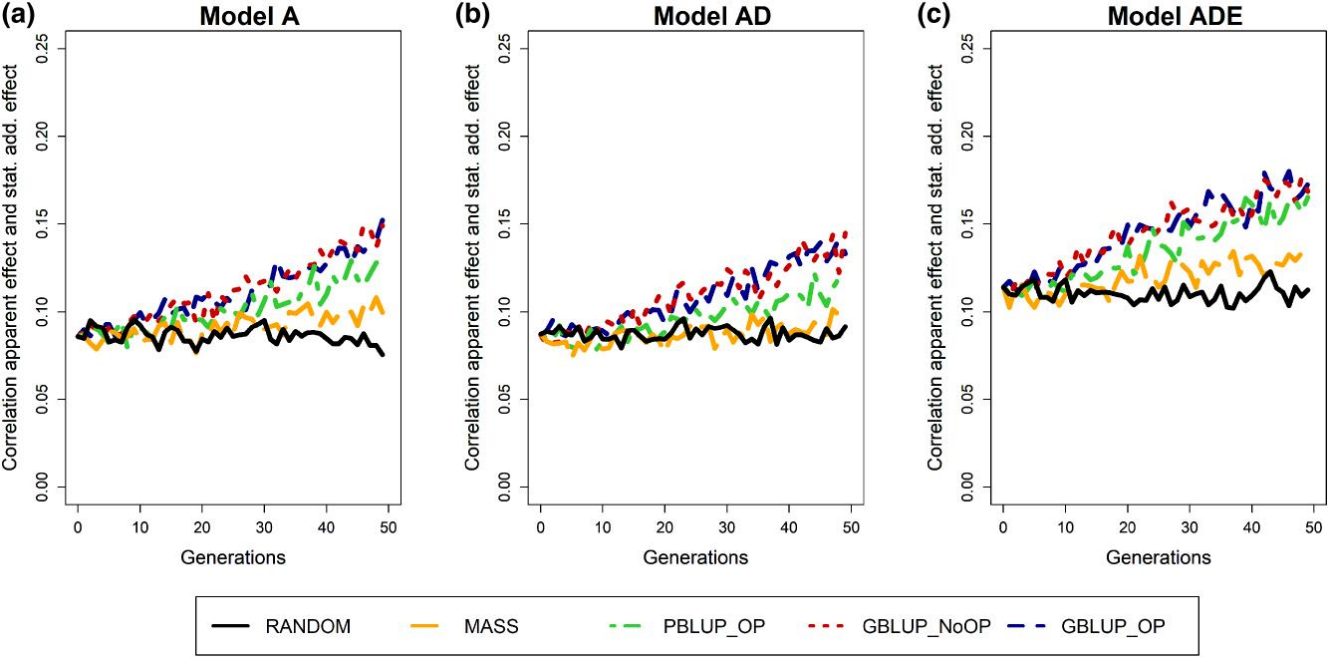
Correlation between the apparent effect and the statistical additive effect of causal loci across generations for the 5 selection methods and 3 genetic models. The 5 selection methods were as follows: RANDOM selection, MASS selection, PBLUP selection with own performance (PBLUP_OP), GBLUP selection without own performance (GBLUP_NoOP) or with own performance (GBLUP_OP). The 3 genetic models were a model with a) only additive effects (A), with b) additive and dominance effects (AD), or with c) additive, dominance, and epistatic effects (ADE). Results are shown as averages of 20 replicates.

In agreement with our expectations, loci with larger statistical additive effects had on average also larger apparent effects (Supplementary File 5). However, the correlation between the statistical additive effect and the apparent effects within a generation was always below 0.2 (Fig. 2). These results indicate that the apparent effect of a locus is for a larger part determined by the linkage with other loci than by the statistical additive effect. This linkage is partly a result of the LD pattern, but might also be a result of sampling in a small population. When only including loci with a MAF above 0.05, the correlation between the statistical additive effects and apparent effects became stronger (∼0.3 to ∼0.4; Supplementary Fig. 1.9 in Supplementary File 1). This occurs because for loci with a MAF below 0.05, the probability is higher that, just by chance, all individuals carrying the rare allele will have a below or above-average breeding value, thereby generating an apparent effect that is less correlated to the statistical additive effect of the locus. So, the impact of other loci on the apparent effect of a locus is larger when a locus has a lower MAF.

**Fig. 3.**
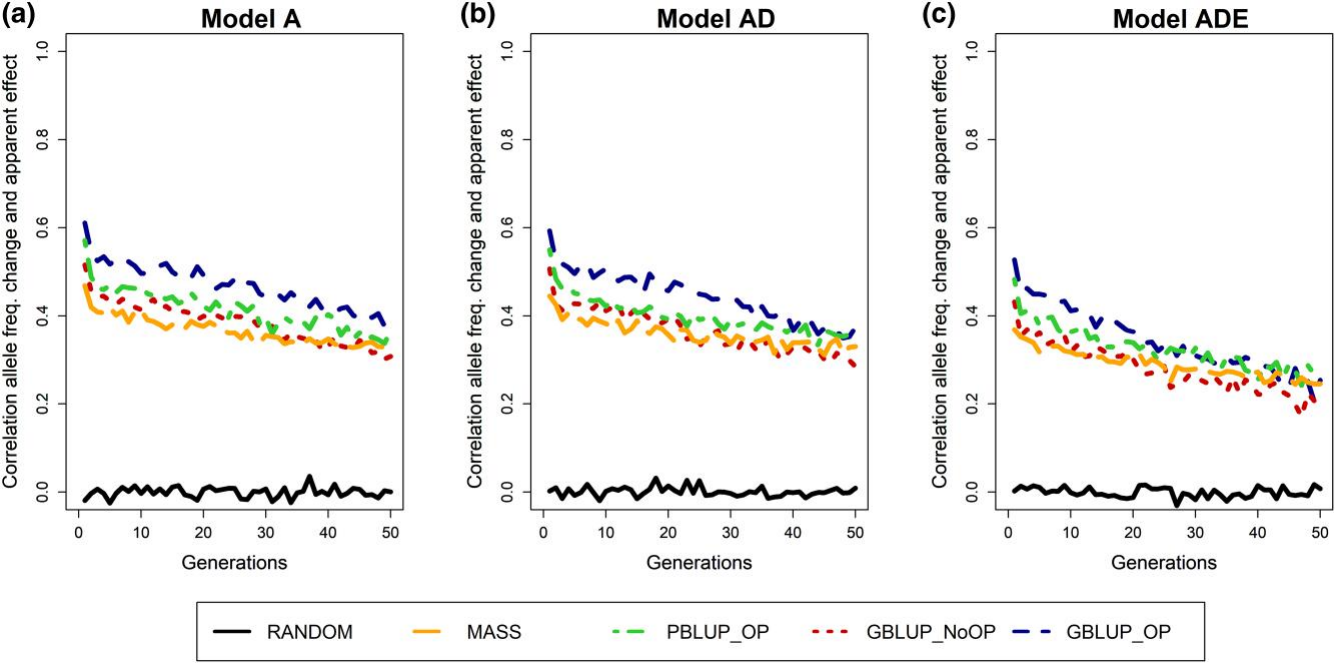
Correlation between the change in allele frequency toward the next generations and the apparent effect of causal loci for the 5 selection methods and 3 genetic models. The change in allele frequency is expressed as the absolute change in allele frequency from generation *i* to generation *i* + 1 divided by *p_i_*(1 − *p_i_*), where *p_i_* is the allele frequency in generation *i*. The 5 selection methods were as follows: RANDOM selection, MASS selection, PBLUP selection with own performance (PBLUP_OP), GBLUP selection without own performance (GBLUP_NoOP) or with own performance (GBLUP_OP). The 3 genetic models were a model with a) only additive effects (A), with b) additive and dominance effects (AD), or with c) additive, dominance, and epistatic effects (ADE). Results are shown as averages of 20 replicates.

The apparent effect of an allele correlated much stronger with allele frequency change than the statistical additive effect (Fig. 3). The correlation between allele frequency change and apparent effect was highest for GBLUP_OP and lowest for MASS in the first generations of selection. As a result of selection, this correlation decreased over generations, which happened at a faster rate for the GBLUP methods than for MASS and PBLUP. Moreover, the correlation was lower when nonadditive effects, especially epistasis, were present, which agrees with the lower obtained selection accuracy when epistasis was present.

### Causal loci becoming fixed

In this section, we more specifically investigate what the characteristics were of the causal loci that changed at least 0.2 in allele frequency and became fixed over 50 generations. With RANDOM, only ∼0.03% (2 out of 6,000) of the loci became fixed (Table 2; Supplementary Fig. 1.10 in Supplementary File 1). Selection drastically increased the number of loci that became fixed; with MASS, this was 2.2% (133 out of 6,000, model A), while it was even higher for PBLUP_OP, GBLUP_NoOP, and GBLUP_OP (∼6%; 357 out of 6,000, model A). The presence of nonadditive effects substantially reduced the number of loci that became fixed (50% lower with MASS and 30% lower with PBLUP_OP, GBLUP_NoOP, and GBLUP_OP for model ADE compared to model A). With MASS, the loci that were fixed were more frequently fixed for the favorable allele (82–89% of the cases) compared to the other selection methods (∼80% under models A and AD and ∼70% under model ADE). For all selection methods, the loci fixed for the favorable allele had an above-average statistical additive effect (Table 2). The average starting allele frequency of the favorable alleles that became fixed was highest for RANDOM (∼0.75), followed by MASS (∼0.61), and lowest for PBLUP_OP, GBLUP_NoOP, and GBLUP_OP (∼0.54), indicating that the average change in allele frequency at loci where an allele became fixed was largest for PBLUP_OP, GBLUP_NoOP, and GBLUP_OP.

**Table 2.** Characteristics of causal loci that became fixed*^a^* over 50 generations of selection for the 5 selection methods and 3 genetic models*^b^*.

	Total no.	Prop. Fav.*^e^*	Fixed for favorable allele*^c^*		Fixed for unfavorable allele*^d^*	
			Avg. *α*/*σ*_G_ in gen. 0*^f^*	Start Afreq	Avg. *α*/*σ*_G_ in gen. 0*^f^*	Start Afreq
Model A						
RANDOM	2 (0.4)	0.46 (0.09)	0.035 (0.0046)	0.74 (0.010)	0.046 (0.0067)	0.25 (0.011)
MASS	133 (5.3)	0.89 (0.01)	0.061 (0.0008)	0.59 (0.004)	0.021 (0.0011)	0.34 (0.009)
PBLUP_OP	358 (4.6)	0.80 (0.01)	0.053 (0.0005)	0.52 (0.003)	0.025 (0.0006)	0.39 (0.004)
GBLUP_NoOP	356 (5.7)	0.79 (0.00)	0.052 (0.0005)	0.52 (0.003)	0.027 (0.0006)	0.40 (0.005)
GBLUP_OP	357 (5.3)	0.82 (0.00)	0.054 (0.0005)	0.51 (0.003)	0.024 (0.0006)	0.40 (0.005)
Model AD						
RANDOM	2 (0.4)	0.59 (0.09)	0.030 (0.0057)	0.74 (0.012)	0.045 (0.0078)	0.24 (0.012)
MASS	103 (3.4)	0.88 (0.01)	0.056 (0.0008)	0.61 (0.004)	0.020 (0.0011)	0.32 (0.007)
PBLUP_OP	289 (6.7)	0.81 (0.01)	0.051 (0.0005)	0.54 (0.003)	0.024 (0.0006)	0.37 (0.005)
GBLUP_NoOP	328 (5.3)	0.78 (0.00)	0.050 (0.0005)	0.52 (0.003)	0.026 (0.0006)	0.40 (0.005)
GBLUP_OP	319 (4.2)	0.82 (0.01)	0.051 (0.0005)	0.52 (0.003)	0.024 (0.0006)	0.38 (0.005)
Model ADE						
RANDOM	3 (0.3)	0.45 (0.09)	0.017 (0.0040)	0.75 (0.009)	0.030 (0.0056)	0.25 (0.008)
MASS	64 (2.4)	0.82 (0.01)	0.052 (0.0015)	0.65 (0.004)	0.012 (0.0009)	0.33 (0.008)
PBLUP_OP	248 (4.0)	0.69 (0.01)	0.034 (0.0007)	0.58 (0.003)	0.014 (0.0004)	0.38 (0.004)
GBLUP_NoOP	256 (6.4)	0.68 (0.01)	0.034 (0.0006)	0.58 (0.003)	0.014 (0.0004)	0.40 (0.004)
GBLUP_OP	250 (5.5)	0.71 (0.01)	0.035 (0.0007)	0.57 (0.003)	0.013 (0.0004)	0.40 (0.005)

The 5 selection methods were as follows: RANDOM selection, MASS selection, PBLUP selection with own performance (PBLUP_OP), GBLUP selection without own performance (GBLUP_NoOP) or with own performance (GBLUP_OP). The 3 genetic models were a model with only additive effects (A), with additive and dominance effects (AD), or with additive, dominance, and epistatic effects (ADE).

The minimum change in allele frequency was set at 0.2.

Results are shown as averages across replicates with their corresponding standard errors of the mean between brackets.

Loci fixed for the favorable allele, based on the statistical additive effect in generation 0.

Loci fixed for the unfavorable allele, based on the statistical additive effect in generation 0.

Proportion of loci fixed for the favorable allele, based on the statistical additive effect in generation 0.

Average absolute statistical additive effect (*α*) divided by the total genetic standard deviation in generation 0 of loci that became fixed in the population.

Selection also resulted in a substantial fraction (∼10–30%) of the loci that became fixed for the unfavorable allele. The number of loci that became fixed for the unfavorable allele was similar with GBLUP_OP, GBLUP_NoOP, and PBLUP_OP, and roughly 5 times larger than with MASS, while the number of loci that became fixed for the favorable allele was only roughly 3 times larger with GBLUP_OP, GBLUP_NoOP, and PBLUP_OP than with MASS. Moreover, the loci fixed for the unfavorable allele were on average more unfavorable (i.e. had on average a more negative statistical additive effect) with GBLUP_OP, GBLUP_NoOP, and PBLUP_OP than with MASS. For the loci that became fixed for the unfavorable allele, the average frequency of the favorable allele in generation 0 was lowest for RANDOM (∼0.25), followed by MASS (∼0.33), and finally PBLUP_OP, GBLUP_NoOP, and GBLUP_OP (∼0.39). This means that, similar to the results for the loci fixed for the favorable allele, the average change in allele frequency at loci where an allele became fixed was largest for PBLUP_OP, GBLUP_NoOP, and GBLUP_OP, followed by MASS and finally RANDOM.

### Fixation of causal loci due to selection vs drift

**Fig. 4.**
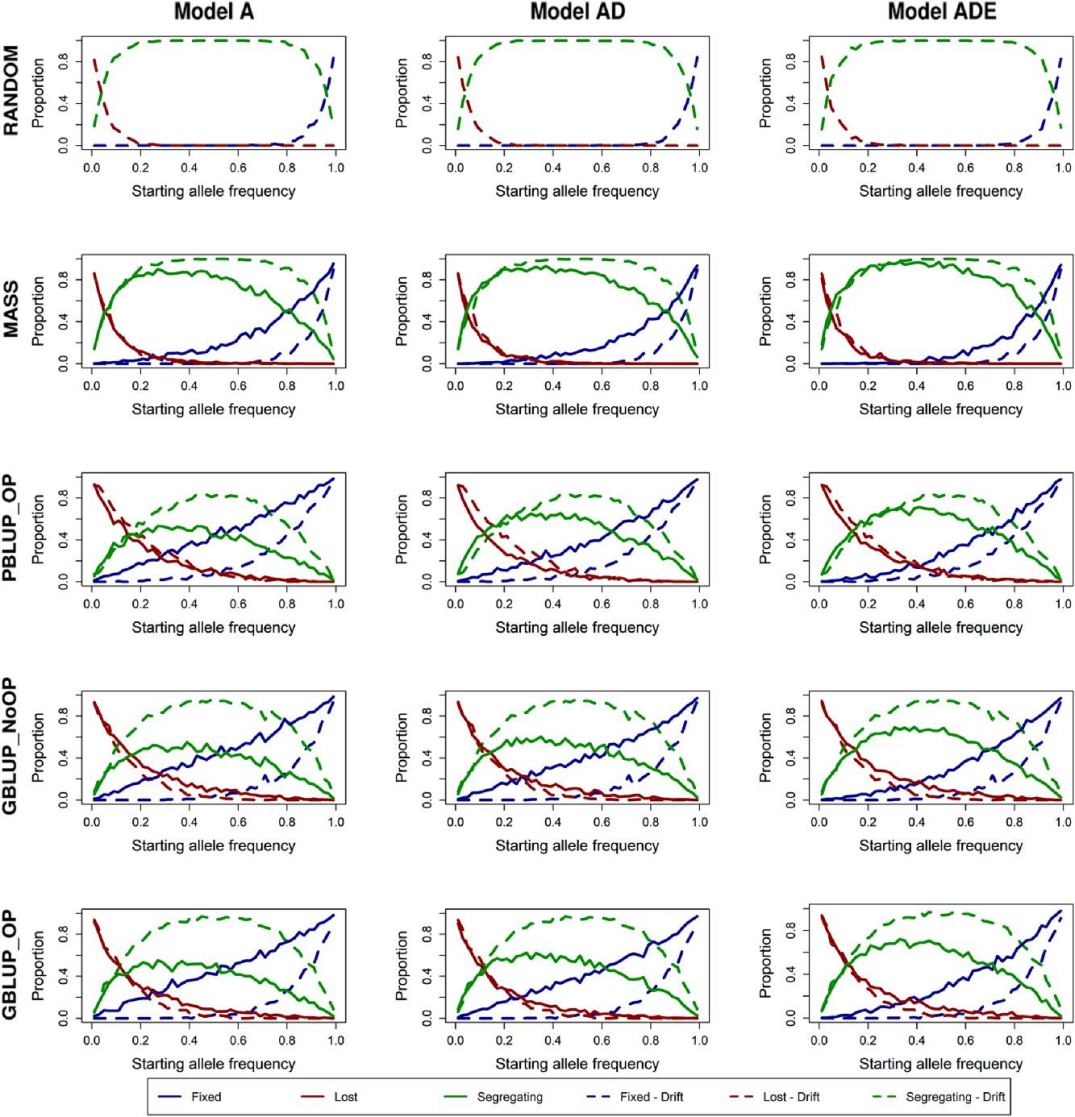
Proportion of loci with a given starting allele frequency (generation 0) that became either fixed, lost, or remain segregating after 50 generations of selection vs 50 generations of drift for the 5 selection methods and 3 genetic models. A locus was set to be fixed when the favorable allele was fixed and lost when the favorable allele was lost, where the favorable allele was assigned based on the statistical additive effect in generation 0. The 5 selection methods were as follows: RANDOM selection, MASS selection, PBLUP selection with own performance (PBLUP_OP), GBLUP selection without own performance (GBLUP_NoOP) or with own performance (GBLUP_OP). The 3 genetic models were a model with only additive effects (A), with additive and dominance effects (AD), or with additive, dominance, and epistatic effects (ADE). Results are shown as averages of 20 replicates.

The aim of this section was to investigate how many additional causal loci became fixed due to selection compared to a population with the same *N*_e_ experiencing only drift. The average *N*_e_, calculated from pedigree kinship, was largest for RANDOM (222), followed by MASS (143), GBLUP_OP (81), and GBLUP_NoOP (72), and lowest for PBLUP_OP (47; Table 3). As expected, with drift alone, the highest probability of fixation or loss of the favorable allele was achieved with the lowest *N*_e_ (PBLUP) and the lowest probability with the highest *N*_e_ (RANDOM; Fig. 4). With drift alone, the probability of fixation or loss was approximately equal and depended only on the starting allele frequency and was independent of its effect for all genetic models. Selection resulted in a much higher proportion of favorable alleles being fixed than unfavorable alleles, especially at intermediate and higher starting frequencies and for loci with an above-average statistical additive effect. Moreover, selection resulted in a lower probability of loci to remain segregating.

**Table 3.** Proportion (in percentage) of causal loci segregating in generation 0 that became fixed for the favorable or unfavorable allele as a result of selection and drift for the 5 selection methods and 3 genetic models.

	Effective population size*^a^*	Proportion of loci fixed for favorable allele (in %)			Proportion of loci fixed for unfavorable allele (in %)		
		Simulation: drift + selection	Drift*^b^*	Selection only	Simulation: drift + selection	Drift*^b^*	Selection only
Model A							
RANDOM	222 (0.8)	8.7	8.7	0.0	8.5	8.5	0.0
MASS	138 (1.7)	29.0	10.7	18.3	12.7	10.6	2.0
PBLUP_OP	47 (0.9)	46.1	21.4	24.7	23.2	22.0	1.2
GBLUP_NoOP	76 (1.4)	45.2	15.2	30.0	23.4	15.9	7.5
GBLUP_OP	83 (1.4)	46.2	14.8	31.4	21.4	15.5	5.9
Model AD							
RANDOM	222 (0.8)	8.5	8.5	0.0	8.5	8.5	0.0
MASS	144 (1.3)	24.7	10.7	14.0	11.3	10.6	0.6
PBLUP_OP	53 (1.1)	40.5	21.4	19.1	20.0	22.0	−2.0
GBLUP_NoOP	75 (1.3)	42.0	15.2	26.9	22.0	15.9	6.1
GBLUP_OP	87 (1.0)	42.6	14.8	27.7	19.7	15.5	4.3
Model ADE							
RANDOM	222 (1.0)	8.2	8.2	0.0	8.8	8.8	0.0
MASS	151 (1.4)	20.6	10.7	9.9	11.2	10.6	0.5
PBLUP_OP	44 (0.9)	35.0	21.4	13.6	23.3	22.0	1.3
GBLUP_NoOP	69 (1.0)	35.0	15.2	19.8	24.0	15.9	8.2
GBLUP_OP	76 (1.1)	35.2	14.8	20.3	22.4	15.5	6.9

The 5 selection methods were as follows: RANDOM selection, MASS selection, PBLUP selection with own performance (PBLUP_OP), GBLUP selection without own performance (GBLUP_NoOP) or with own performance (GBLUP_OP). The 3 genetic models were a model with only additive effects (A), with additive and dominance effects (AD), or with additive, dominance, and epistatic effects (ADE).

Estimated effective population size based on pedigree inbreeding coefficient with corresponding standard errors across replicates between brackets.

Results from the simulation with only drift, using the average effective population size of the different selection methods across the 3 genetic models, i.e. MASS: 143; PBLUP_OP: 47; GBLUP_NoOP: 72; and GBLUP_OP: 81.

A quantitative analysis shows that, in general, more loci became fixed for the favorable allele due to selection than due to drift (Table 3). Under MASS, an additional ∼14% of the loci (averaged across genetic models A, AD, and ADE) segregating in generation 0 became fixed due to selection compared to drift alone. With PBLUP and GBLUP, this proportion was larger, namely ∼19% and ∼26%, respectively. Nonadditive effects reduced the impact of selection on the fixation of loci. For MASS and PBLUP_OP, the loss of favorable alleles (or fixation of the unfavorable allele) was mainly due to drift and not a result of selection, while approximately an additional 6–7% of loci became lost as a result of selection compared to drift with GBLUP_OP and GBLUP_NoOP.

### Allele frequency changes of new causal mutations

In this last section, we investigate what happened to the new mutations that occurred during the 50 generations of selection. The RANDOM scenario resulted in the highest number of mutants that occurred between generations 1 and 49 and that were still segregating in generation 50, followed by MASS, then by GBLUP_OP and GBLUP_NoOP, and finally by PBLUP_OP (Table 4). For PBLUP_OP, the number of segregating mutants was only ∼45% of that with RANDOM and ∼81% of that with GBLUP_OP and GBLUP_NoOP. The average MAF of the segregating mutants was largest for PBLUP_OP and lowest for RANDOM. No clear trend was observed for the variation in MAF of segregating mutants across the different scenarios.

**Table 4.** Characteristics of causal mutations that were segregating in generation 50*^a^* for the 5 selection methods and 3 genetic models*^b^*.

	Segregating mutations		
	Total no.*^c^*	Avg. MAF*^d^*	Var. MAF*^e^*
Model A			
RANDOM	575 (6.3)	0.019 (0.000)	0.001 (0.0000)
MASS	491 (6.3)	0.029 (0.001)	0.003 (0.0002)
PBLUP_OP	251 (4.2)	0.045 (0.001)	0.004 (0.0013)
GBLUP_NoOP	324 (3.7)	0.030 (0.001)	0.006 (0.0003)
GBLUP_OP	320 (4.5)	0.035 (0.001)	0.008 (0.0002)
Model AD			
RANDOM	580 (4.8)	0.019 (0.000)	0.001 (0.0000)
MASS	532 (5.1)	0.034 (0.001)	0.005 (0.0002)
PBLUP_OP	276 (5.0)	0.051 (0.001)	0.012 (0.0004)
GBLUP_NoOP	320 (4.6)	0.034 (0.001)	0.007 (0.0003)
GBLUP_OP	316 (5.8)	0.042 (0.001)	0.005 (0.0003)
Model ADE			
RANDOM	582 (6.4)	0.018 (0.000)	0.001 (0.0000)
MASS	516 (6.5)	0.031 (0.001)	0.005 (0.0002)
PBLUP_OP	254 (4.1)	0.050 (0.001)	0.010 (0.0003)
GBLUP_NoOP	321 (5.4)	0.033 (0.001)	0.004 (0.0003)
GBLUP_OP	320 (4.1)	0.039 (0.001)	0.008 (0.0004)

The 5 selection methods were as follows: RANDOM selection, MASS selection, PBLUP selection with own performance (PBLUP_OP), GBLUP selection without own performance (GBLUP_NoOP) or with own performance (GBLUP_OP). The 3 genetic models were a model with only additive effects (A), with additive and dominance effects (AD), or with additive, dominance, and epistatic effects (ADE).

Only mutations introduced between generations 1 and 49 were considered, not the new mutations in generation 50.

Results are shown as averages across replicates with their corresponding standard errors of the mean between brackets.

Total number of mutations segregating in generation 50.

Average MAF of mutations segregating in generation 50.

Variation in MAF of mutations segregating in generation 50.

**Fig. 5.**
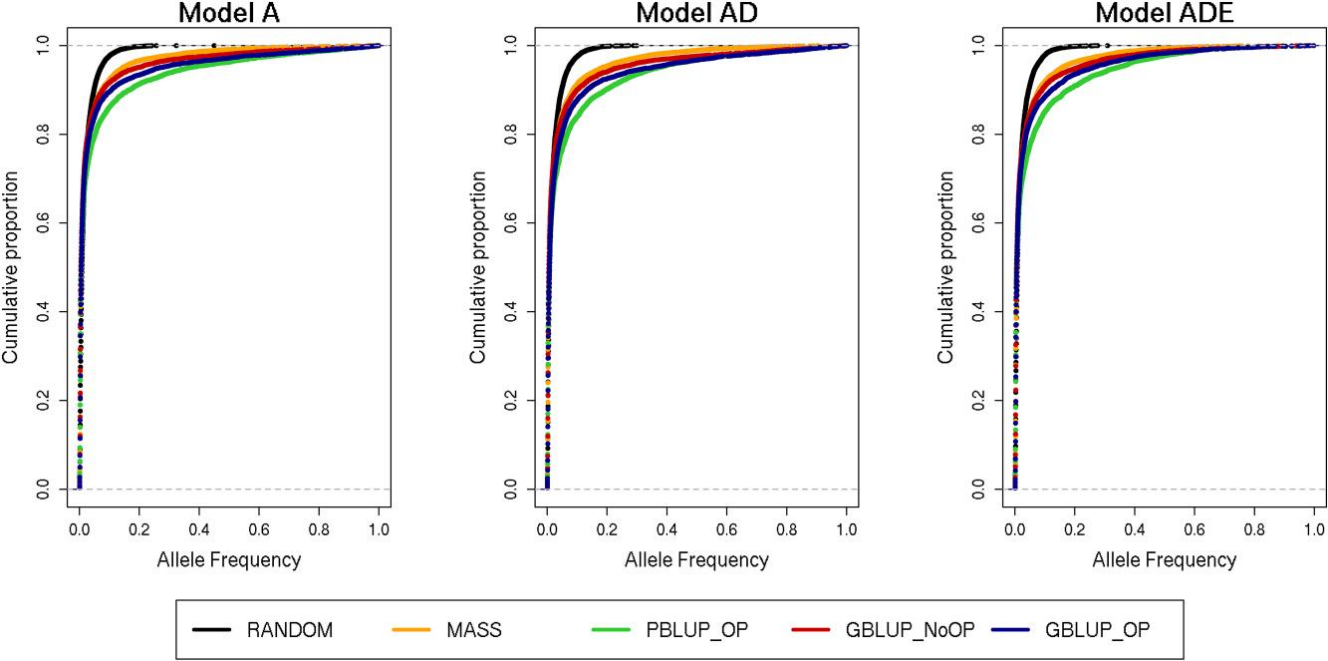
Cumulative distribution function of the allele frequency of the mutated allele in generation 50 of causal mutations introduced between generations 1 and 49 for the 5 selection methods and 3 genetic models. The 5 selection methods were as follows: RANDOM selection, MASS selection, PBLUP selection with own performance (PBLUP_OP), GBLUP selection without own performance (GBLUP_NoOP) or with own performance (GBLUP_OP). The 3 genetic models were a model with only additive effects (A), with additive and dominance effects (AD), or with additive, dominance, and epistatic effects (ADE). The cumulative distribution function is made across all 20 replicates.

More than 90% of the segregating mutants had a frequency below 0.1 across scenarios, except for PBLUP_OP, where this proportion was slightly lower (Fig. 5). For RANDOM, the maximum allele frequency of a segregating mutant was around 0.4. Over 50 generations of MASS, the maximum allele frequency of a mutant was 0.988. With PBLUP_OP, GBLUP_NoOP, and GBLUP_OP, some mutants became fixed (Supplementary Table 2.4 in Supplementary File 2). On average, ∼40% more mutants became fixed with GBLUP_OP than with PBLUP_OP and ∼85% less mutants when epistatic effects were present. However, the number of mutants becoming fixed was in general limited for all scenarios.

## Discussion

The aim of this study was to compare random, phenotypic, pedigree, and genomic selection with respect to changes in allelic architecture of existing and new causal loci over 50 generations of selection. Moreover, we investigated whether the impact of these selection methods depended on the genetic architecture of the trait, by investigating traits determined by only additive, additive and dominance, or additive, dominance, and epistatic effects. Since the vast majority of causal loci and their interactions are unknown in empirical data, we used simulations. We extend beyond previous work by including the impact of nonadditive effects on changes in allelic architecture and by considering a long time horizon. A better understanding of the changes in allelic architecture under selection can help to better predict the long-term effects of selection and may provide clues on how to optimize selection methods to limit undesired loss of genetic variation.

### Changes in allelic architecture of causal loci

As expected, the change in allele frequency was larger for the methods with a higher selection accuracy, while the presence of epistatic effects resulted in a lower allele frequency change. The lower allele frequency change with epistasis is partly a result of a lower narrow-sense heritability (∼0.25 for model ADE compared to ∼0.38 for model AD and ∼0.4 for model A), which resulted in a lower selection accuracy. Moreover, epistasis changed the statistical additive effects of loci over generations due to changes in allele frequencies, which also changed the pressure and direction of selection (Barton *et al*. 2017; Falconer and Mackay 1996; Legarra *et al*. 2021; Wientjes *et al*. 2022). Those changes in direction of selection limited the total change in allele frequency. Finally, epistasis could have resulted in a lower allele frequency change because it created a negative correlation between statistical additive effect and MAF (Wientjes *et al*. 2022). This negative correlation is in agreement with observations in empirical data (Manolio *et al*. 2009; Marouli *et al*. 2017; Zeng *et al*. 2018) and a result of a larger conversion of nonadditive effects into statistical additive effects when allele frequencies are closer to 0 or 1 (Barton and Turelli 2004; Hill *et al*. 2008; Mäki-Tanila and Hill 2014). This also means that the additive genetic variance explained by a locus, $2p(1-p)\alpha^{2}$, changes with epistasis due to changes in both *p* and *α* (Neiman and Linksvayer 2006; Whitlock *et al*. 1995) and, in our study, was highest for loci with a MAF of ∼0.25. Thus, epistasis can limit the change in allele frequency over time due to a lower narrow-sense heritability, changes in pressure and direction of selection over time, and the negative correlation between MAF and effect size.

Due to the potential changes in selection pressure on loci across generations when epistasis was present (Barton *et al*. 2017), we expected that causal loci involved in more interactions would show a smaller change in allele frequency. Surprisingly, our results showed the opposite, and loci with a larger change in allele frequency were generally involved in more interactions (Supplementary Table 4.3 in Supplementary File 4). This is most likely a result of the positive relation between the number of interactions and the size of the statistical additive effects (Supplementary Fig. 1.11 in Supplementary File 1).

### Fixation of causal loci

With more accurate selection, i.e. going from MASS to GBLUP, more causal loci became fixed, both for the favorable and unfavorable alleles. For the selection scenarios, GBLUP in particular, there was a higher number of loci fixed for the unfavorable allele than expected due to drift alone. This was surprising, since selection was expected to prevent favorable alleles from becoming lost (Robertson 1960; Walsh and Lynch 2018). However, it is also known that unfavorable alleles can get fixed with selection due to genetic hitchhiking (Barton 2000; Bosse *et al*. 2019; Hospital and Chevalet 1996; Smith and Haigh 1974): the rapid increase in frequency of favorable alleles also changes the frequency of unfavorable alleles at linked loci, because recombination can be too slow to break up the link between loci. This effect can be considerable and span a large part of a chromosome (Pedersen *et al*. 2010), and it is desirable but challenging to limit hitchhiking in breeding programs (Sonesson *et al*. 2012). Especially for GBLUP, the number of unfavorable alleles that became fixed was larger than with just drift (Table 3). This indicates that the effect of hitchhiking was largest for GBLUP due to the higher selection pressure, which is in agreement with previous research showing a stronger hitchhiking effect for GBLUP than for PBLUP (Liu *et al*. 2014). Overall, those results indicate that it will be even more important to limit hitchhiking in genomic breeding programs compared to pedigree breeding programs.

The degree of hitchhiking, and thereby the chance of losing favorable alleles, will depend on the genetic architecture of the trait, where fewer mutations of larger effect will result in more hitchhiking. Hence, the practical relevance of hitchhiking will critically depend on the true genetic architecture, of which we still know little. However, in actual breeding programs, selection usually takes place on multiple traits and is therefore most likely influenced by many causal loci of which some might have antagonistic pleiotropic effects. Therefore, the impact of hitchhiking can be expected to be lower in practical scenarios compared to our simulations.

One way to reduce the fixation of unfavorable alleles is to reduce the loss of rare favorable alleles by increasing the weight of those alleles in the selection index (Goddard 2009). This approach can also be applied to markers and can increase long-term genetic gain and maintain more genetic variance, but at the expense of short-term genetic gain (De Beukelaer *et al*. 2017; Jannink 2010; Liu *et al*. 2015). More research is still needed to find the optimal weights for rare alleles while minimizing the loss in short-term gain.

### Apparent effects

The change in allele frequency from 1 generation to the next was only very weakly related to the statistical additive effect of the locus (Supplementary Fig. 1.8 in Supplementary File 1), while the correlation was much higher with the apparent effect (Fig. 3). This shows that other loci have a large impact on the temporal selection pressure on a locus.

The apparent effects as estimated in our study are based on the true breeding values (i.e. total additive genetic value) of the individuals, which are generally unknown, and actual selection takes place based on estimated breeding values. Therefore, we also estimated the apparent effects based on the estimated breeding values as the *simple* regression of the estimated breeding values on allele counts of a causal locus. The correlation with the change in allele frequency was very similar across selection methods and much higher (∼0.7; Supplementary Fig. 1.12 in Supplementary File 1) than for the apparent effects based on true breeding values (Fig. 3). This high correlation indicates that the impact of selection on the allele frequency change is affected by the LD with other causal loci as well as the accuracy of estimating the effect of the locus (Walsh and Lynch 2018).

### New causal mutations

New causal mutations will usually be lost by drift within a few generations, largely independent of their effect (Walsh and Lynch 2018). Only when the mutant reaches a meaningful allele frequency, selection starts to have an effect (Eyre-Walker 2010; Lande 1983; Robertson 1960; Walsh and Lynch 2018). As expected based on previous research (Hill 1982b; Weber and Diggins 1990), our results showed that the selection method with the largest *N*_e_ (MASS) maintained the most mutations over 50 generations of selection, followed by GBLUP and finally PBLUP, which had the lowest *N*_e_. The average MAF of the maintained mutants was highest for PBLUP, followed by GBLUP and finally MASS, which agrees with previous observations for mutations (Mulder *et al*. 2019) and segregating causal loci under selection (Wientjes *et al*. 2022).

The genic variance created by the segregating mutations was largest with MASS, followed by GBLUP_OP and PBLUP_OP, and finally GBLUP_NoOP (Supplementary Fig. 1.13 in Supplementary File 1). This is in agreement with results of Mulder *et al*. (2019), who showed that for maintaining mutational genic variance, OP records of the selection candidates are important. However, actual breeding programs select for multiple traits, and OP records are typically unavailable for at least 1 of those traits. Typical examples are milk yield, litter size, egg number, and carcass traits in animal breeding and traits measured on hybrids in plant breeding. Thus, selection schemes based solely on OP information are not feasible in practice in most cases. Interestingly, however, the difference in mutational genic variance between genomic selection with and without OP was smaller when epistatic effects were present, especially in the last generations. This could be a result of the lower narrow-sense heritability for this genetic model (∼0.25 compared to ∼0.38 for model AD and ∼0.4 for model A; Wientjes *et al*. 2022), which puts less emphasis on OP records in the breeding value estimation (Falconer and Mackay 1996).

### Relevance for practical breeding programs

The aim of our simulations was to compare the long-term impact of different selection methods relevant to livestock and plant breeding on allelic architecture. When simulating the breeding program, it was necessary to make several simplifying assumptions. Here, we consider the likely impact of these assumptions on the comparison of the selection methods for their change in allelic architecture in a practical breeding program.

The first assumption is that we focus only on a single trait, while in practical breeding programs, selection takes place on an index combining multiple traits. The number of causal loci underlying the index is generally larger than in our simulations, and loci can have antagonistic pleiotropic effects on the different traits in the selection index. This can reduce the selection pressure on each locus, but not the ranking of the selection methods in terms of their impact on allelic architecture. Moreover, the weights of the different traits in the index can change over time or new traits can be added. This can also change the selection pressure and direction on a locus over time, which is in a way comparable to our scenarios with epistasis. Given that the presence of epistasis only reduced the absolute differences between the selection methods, but not the ranking, we expect that a changing selection index will not result in reranking of the methods.

The second assumption is that we used the same marker panel across all generations for the GBLUP scenarios. A high proportion of those markers (>50%) was fixed in generation 50 (Supplementary Fig. 1.3 in Supplementary File 1). In reality, the fixation of markers would probably result in updating the marker panel, for example by replacing the fixed markers by segregating markers or by adding identified causal variants to the chip. This might increase the accuracy of selection for the GBLUP scenarios in later generations, but not for the other scenarios that are not based on the marker panel. Therefore, we do not expect a large impact of this on the ranking of the selection methods in terms of changing the allelic architecture. Moreover, the higher number of loci underlying the selection index in practical breeding programs compared to our simulations is also expected to reduce the allele frequency change at the markers, which might also reduce the need to update the marker panels for genomic selection.

The third assumption is related to the structure of the population, where we used in every discrete generation 100 dams and 100 sires with a mating ratio of 1:1 and a litter size of 10. Differences in population structure are reflected in the *N*_e_ of the population, which was in our case comparable to the *N*_e_ observed in livestock populations (Hall 2016). The value of *N*_e_ describes the level of drift in the population (Falconer and Mackay 1996). In our simulations, the different selection methods resulted in different values of *N*_e_ due to differences in inbreeding. In practice, however, many breeding programs use optimal contribution selection (Meuwissen 1997) and optimize the breeding program to obtain a pre-set value for *N*_e_, which is often set to the same value regardless of the selection method used. At the moment, it is difficult to predict how the selection methods would rank when the same *N*_e_ value is used for each selection method.

The fourth assumption is related to the mutation pattern. The actual pattern of mutations affecting traits under selection in livestock populations is largely unknown. Therefore, we decided to use the same distribution for sampling functional additive, dominance, and epistatic effects for mutations as we did for causal loci and to base the average number of mutations per individual (0.6) on the number of mutations needed to reach a mutational variance of ∼0.001$\sigma_{e}^{2}$, the value that is generally reported in experimental populations (Hill 1982a; Houle *et al*. 1996; Lynch and Walsh 1998). Mutations were favorable in approximately 50% of cases. This might be higher than the actual percentage of favorable mutations observed for fitness traits (Bataillon and Bailey 2014; Eyre-Walker and Keightley 2007; Sanjuán *et al*. 2004); however, for other traits, not much is known about this percentage. The rate of de novo mutations used for the simulated trait was lower than that reported for human newborns, which ranges from 74 to 100 (Campbell and Eichler 2013; Lindsay *et al*. 2019; Lynch 2016; Veltman and Brunner 2012). However, not all de novo mutations have an effect on the trait of interest, and we only simulated mutations that affected the trait. Moreover, the actual number of causal loci underlying a trait in actual livestock populations is probably much higher than in our simulations, and it is the proportion of new mutations relative to the proportion of segregating causal loci that affects the mutational variance, not the number of new mutations per se. More importantly, it has been shown before that assumptions about the mutational variance and distribution of mutational effects can impact the contribution of new mutations to long-term genetic gain and variance; however, they did not impact the ranking of selection methods (Mulder *et al*. 2019).

## Conclusion

Altogether, our results show that genomic selection results in slightly larger and faster changes in allelic architecture of causal loci than pedigree selection and much larger and faster changes than phenotypic selection. The presence of nonadditive effects limits the change in allele frequency, because nonadditive effects can change the selection pressure and direction on a locus over generations. Loci with a larger statistical additive effect change on average more in allele frequency over 50 generations. However, the selection pressure on a locus is also largely affected by the linkage phase with other loci and by the accuracy by which the effect can be estimated.

Genomic and pedigree selection fixed in general more loci after 50 generations, and in particular much more unfavorable alleles, than phenotypic selection. This increase was more related to hitchhiking with genomic selection compared to pedigree selection, showing the importance to minimize hitchhiking in genomic breeding programs to limit the loss of favorable alleles that are important for long-term genetic improvement.

Phenotypic selection was best in creating new genetic variance by maintaining mutations, followed by genomic and pedigree selection with OP records, and finally genomic selection without OP records. This shows that OP records are important to use in selection to optimally exploit new mutations that are essential for long-term genetic gain.

## Supplementary Material

iyad141_Supplementary_Data

## Data Availability

Supplementary File 6 contains the QMSim input file, Fortran programs, and seeds used to select the markers and causal loci, to simulate functional effects, genotypes and phenotypic values of new generations, and the interaction matrix used to simulate epistatic effects. Supplemental material available at GENETICS online.
