# Supplementary material for: The long-term effects of genomic selection: 2. Changes in allele frequencies of causal loci and new mutations
Source: Genetics. 2023 Jul 28;225(1):iyad141. doi: 10.1093/genetics/iyad141 (PMC10471209; doi:10.1093/genetics/iyad141)
Supplement: iyad141_Supplementary_Data [file iyad141_supplementary_data.zip › File_S4_GENETICS-2023-306366.docx]

**File S4: Characteristics of loci with a large or small change in allele frequency**

For RANDOM, there were no causal loci with a large (>0.9) change in allele frequency (Figure S4.1, Table S4.1, Table S4.2). With MASS, only few causal loci (≤0.1%; ≤6 out of 6000 loci) had a large change in allele frequency. With the other selection methods, ~0.3% (17 out of 6000) to ~0.5% (31 out of 6000) of the causal loci showed a large change in allele frequency under both model A and AD, and ~0.1% (8 out of 6000) under model ADE. Across all genetic models, the GBLUP methods resulted in ~24% more loci with a large change in allele frequency than PBLUP_OP. With MASS selection, almost all loci with a large change in allele frequency were changed in the favorable direction (~99%). This was not the case for the other selection methods, where the frequency of the unfavorable allele increased by more than 0.9 for 2-19% of the loci, especially when epistasis was present.

On average, the loci with a large change in allele frequency had a larger functional additive and dominance effect than an average locus (Table S4.1). Moreover, the loci with a large allele frequency change had a larger statistical additive effect in generation 0 for all genetic models. This was most pronounced with MASS, followed by PBLUP_OP and GBLUP_OP, and least pronounced with GBLUP_NoOP. Moreover, the loci with a large change in allele frequency were on average more involved in interactions compared to an average locus (Table S4.3).

Only few loci (0.1 – 1%) showed a small change in allele frequency (<0.01) over 50 generations (Table 2). Even though the change in allele frequency from generation 0 to 50 was small, allele frequencies fluctuated considerably (Figure S4.2). This result indicates that an allele frequency change <0.01 is largely due to chance, which agrees with the relatively small functional dominance effects for those loci (Table S4.1), indicating a very limited role for overdominance as a mechanism preventing change in allele frequency.


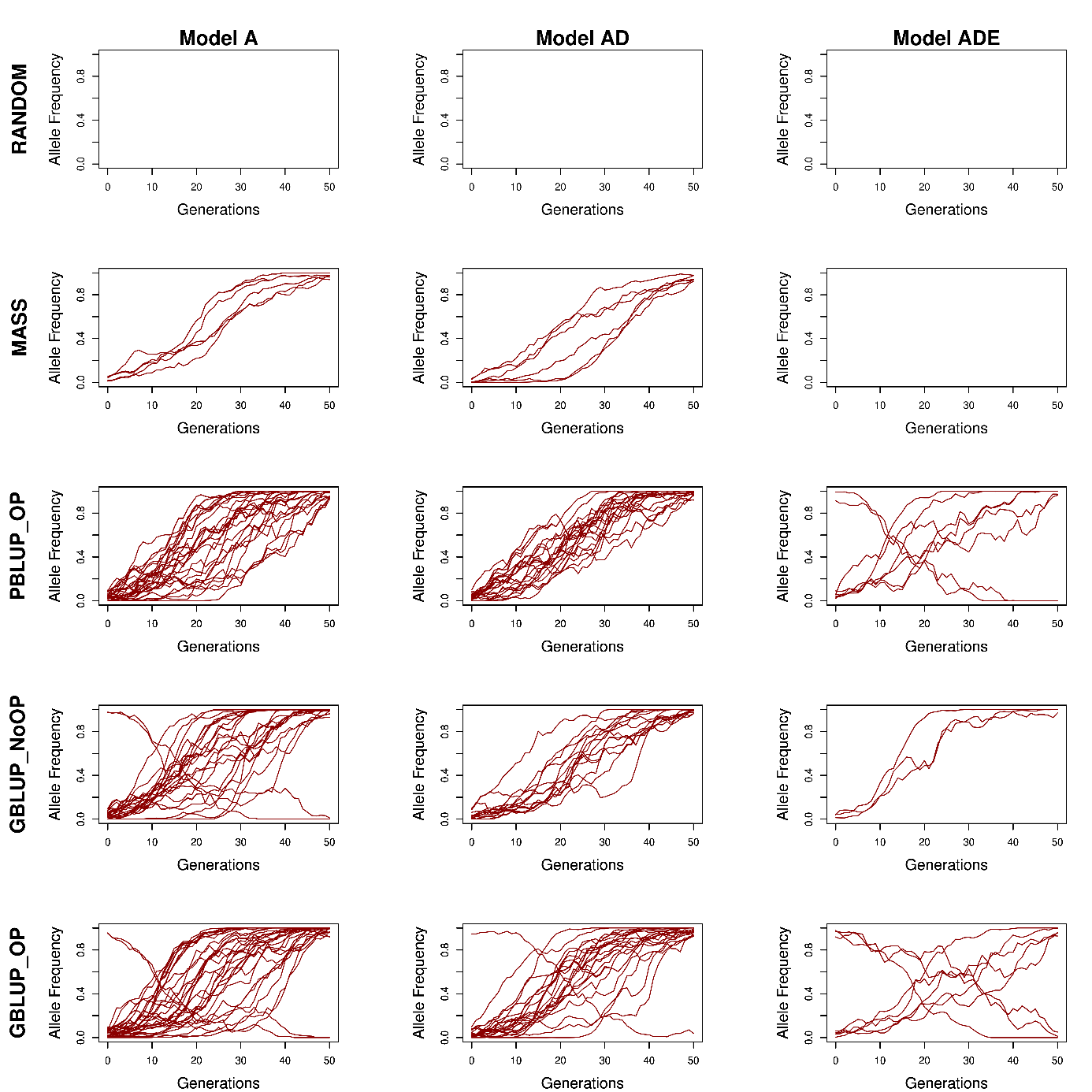


**FIGURE S4.1**

Trend in allele frequency of loci with a large change in allele frequency (>0.9) over the 50 generations of selection for the five selection methods and three genetic models. For all loci, the positive allele was counted, based on the statistical additive effect in generation 0. The five selection methods were: RANDOM selection, MASS selection, PBLUP selection with own performance (PBLUP_OP), GBLUP selection without own performance (GBLUP_NoOP) or with own performance (GBLUP_OP). The three genetic models were a model with only additive effects (A), with additive and dominance effects (AD), or with additive, dominance and epistatic effects (ADE). Results are shown for one replicate.


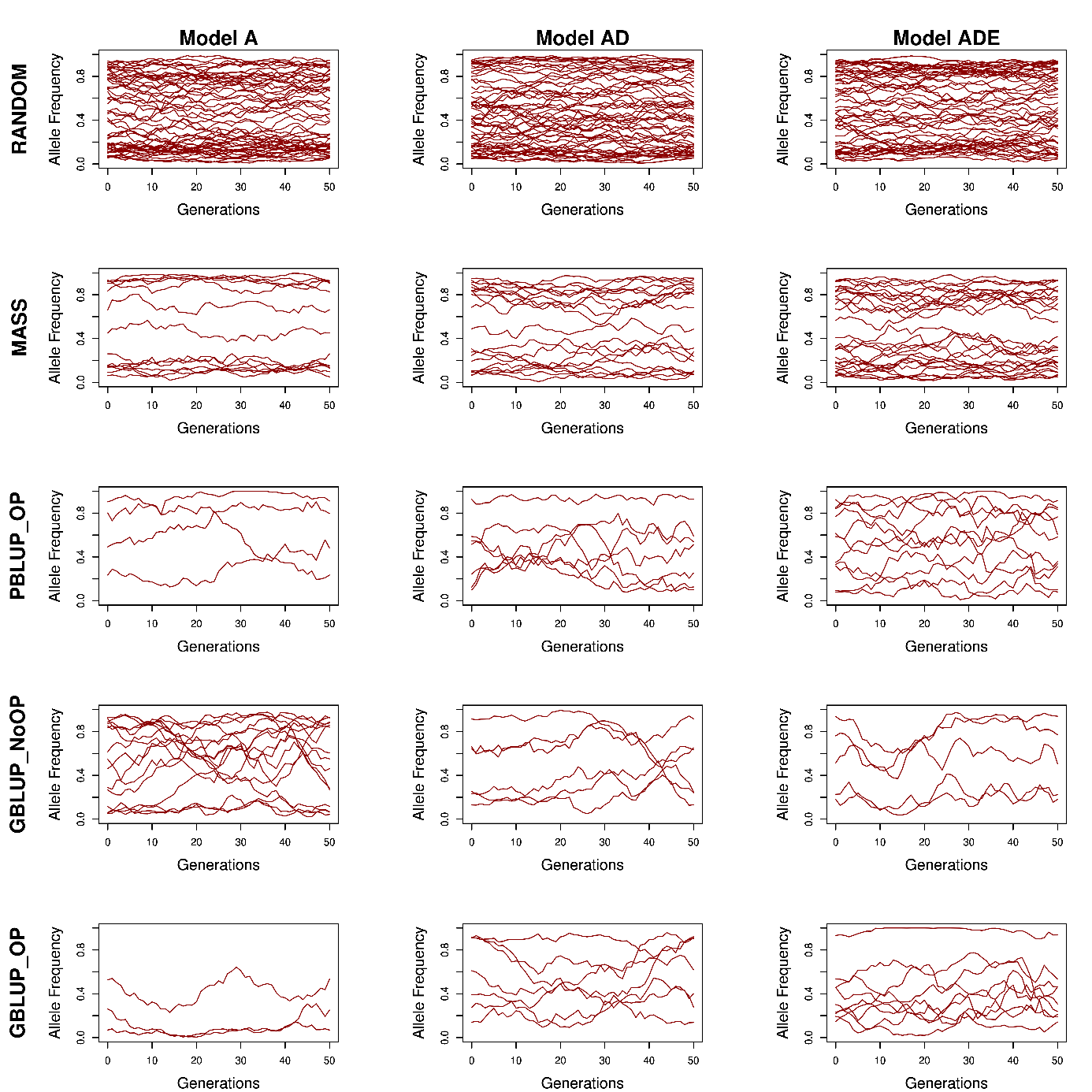


**FIGURE S4.2**

Trend in allele frequency of loci with a small change in allele frequency (<0.01) over the 50 generations of selection for the five selection methods and three genetic models. For all loci, the positive allele was counted, based on the statistical additive effect in generation 0. The five selection methods were: RANDOM selection, MASS selection, PBLUP selection with own performance (PBLUP_OP), GBLUP selection without own performance (GBLUP_NoOP) or with own performance (GBLUP_OP). The three genetic models were a model with only additive effects (A), with additive and dominance effects (AD), or with additive, dominance and epistatic effects (ADE). Results are shown for one replicate.

**TABLE S4.1 –** Characteristics of loci with a large (>0.9) or small (<0.01) change in allele frequency across 50 generations of selection for the five selection methods and three genetic models^1^. The five selection methods were: RANDOM selection, MASS selection, PBLUP selection with own performance (PBLUP_OP), GBLUP selection without own performance (GBLUP_NoOP) or with own performance (GBLUP_OP). The three genetic models were a model with only additive effects (A), with additive and dominance effects (AD), or with additive, dominance and epistatic effects (ADE).

|  | **All loci** | | | |  | **Loci with large (>0.9) change in allele frequency** | | | | |  | **Loci with small (<0.01) change in allele frequency** | | | |
| --- | --- | --- | --- | --- | --- | --- | --- | --- | --- | --- | --- | --- | --- | --- | --- |
|  | **Total Nr.^2^** | **Avg. add. effect^3^** | **Avg. Dom. effect^4^** | **Avg. α/σ_G_ in gen. 0^5^** |  | **Total Nr.^2^** | **Prop. increase favorable allele** | **Avg. add. effect^3^** | **Avg. dom. effect^4^** | **Avg. α/σ_G_ in gen. 0^5^** |  | **Total Nr.^2^** | **Avg. add. effect^3^** | **Avg. dom. effect^4^** | **Avg. α/σ_G_ in gen. 0^5^** |
| ***Model A*** |  |  |  |  |  |  |  |  |  |  |  |  |  |  |  |
| **RANDOM** | 6000 | 0.80 | - | 0.041 |  | 0 | - | - | - | - |  | 63 | 0.83 | - | 0.042 |
| **MASS** | 6000 | 0.80 | - | 0.041 |  | 6 | 0.99 | 1.92 | - | 0.098 |  | 18 | 0.56 | - | 0.029 |
| **PBLUP_OP** | 6000 | 0.80 | - | 0.041 |  | 24 | 0.98 | 1.52 | - | 0.077 |  | 7 | 0.66 | - | 0.033 |
| **GBLUP_NoOP** | 6000 | 0.80 | - | 0.041 |  | 25 | 0.93 | 1.30 | - | 0.066 |  | 7 | 0.58 | - | 0.029 |
| **GBLUP_OP** | 6000 | 0.80 | - | 0.041 |  | 31 | 0.96 | 1.47 | - | 0.075 |  | 7 | 0.51 | - | 0.026 |
|  |  |  |  |  |  |  |  |  |  |  |  |  |  |  |  |
| ***Model AD*** |  |  |  |  |  |  |  |  |  |  |  |  |  |  |  |
| **RANDOM** | 6000 | 0.80 | 0.23 | 0.039 |  | 0 | - | - | - |  |  | 65 | 0.79 | 0.24 | 0.038 |
| **MASS** | 6000 | 0.80 | 0.23 | 0.039 |  | 4 | 1.00 | 1.79 | 0.49 | 0.102 |  | 21 | 0.57 | 0.18 | 0.027 |
| **PBLUP_OP** | 6000 | 0.80 | 0.23 | 0.039 |  | 17 | 0.97 | 1.45 | 0.43 | 0.085 |  | 10 | 0.65 | 0.20 | 0.032 |
| **GBLUP_NoOP** | 6000 | 0.80 | 0.23 | 0.039 |  | 23 | 0.92 | 1.27 | 0.37 | 0.072 |  | 7 | 0.66 | 0.20 | 0.032 |
| **GBLUP_OP** | 6000 | 0.80 | 0.23 | 0.039 |  | 26 | 0.96 | 1.46 | 0.43 | 0.084 |  | 7 | 0.58 | 0.19 | 0.028 |
|  |  |  |  |  |  |  |  |  |  |  |  |  |  |  |  |
| ***Model ADE*** |  |  |  |  |  |  |  |  |  |  |  |  |  |  |  |
| **RANDOM** | 6000 | 0.80 | 0.23 | 0.034 |  | 0 | - | - | - |  |  | 65 | 0.80 | 0.23 | 0.027 |
| **MASS** | 6000 | 0.80 | 0.23 | 0.034 |  | 1 | 0.97 | 1.58 | 0.38 | 0.100 |  | 29 | 0.76 | 0.22 | 0.017 |
| **PBLUP_OP** | 6000 | 0.80 | 0.23 | 0.034 |  | 8 | 0.85 | 1.15 | 0.32 | 0.090 |  | 11 | 0.78 | 0.23 | 0.018 |
| **GBLUP_NoOP** | 6000 | 0.80 | 0.23 | 0.034 |  | 8 | 0.81 | 1.11 | 0.32 | 0.075 |  | 11 | 0.73 | 0.23 | 0.021 |
| **GBLUP_OP** | 6000 | 0.80 | 0.23 | 0.034 |  | 9 | 0.81 | 1.16 | 0.32 | 0.087 |  | 11 | 0.81 | 0.23 | 0.017 |

^1^ Results are shown as averages across replicates. For standard errors of the mean, see File S2; Table S2.4.

^2^ Total number of loci.

^3^ Average absolute functional additive effect of loci that became fixed in the population.

^4^ Average absolute functional dominance effect of loci that became fixed in the population.

^5^ Average absolute statistical additive effect (α) divided by the total genetic standard deviation in generation 0 of loci that became fixed in the population.

**Table S4.2 –** Standard errors of the mean across replicates of the characteristics of loci with a large (>0.9) or small (<0.01) change in allele frequency across 50 generations of selection for the five selection methods and three genetic models. The five selection methods were: RANDOM selection, MASS selection, PBLUP selection with own performance (PBLUP_OP), GBLUP selection without own performance (GBLUP_NoOP) or with own performance (GBLUP_OP). The three genetic models were a model with only additive effects (A), with additive and dominance effects (AD), or with additive, dominance and epistatic effects (ADE).

|  | **All loci** | | | |  | **Loci with large (>0.9) change in allele frequency** | | | | |  | **Loci with small (<0.01) change in allele frequency** | | | |
| --- | --- | --- | --- | --- | --- | --- | --- | --- | --- | --- | --- | --- | --- | --- | --- |
|  | **Total Nr.**^1^ | **Avg. add. effect**^2^ | **Avg. Dom. effect**^3^ | **Avg. α/σ_G_ in gen. 0**^4^ |  | **Total Nr.**^1^ | **Prop. increase favorable allele** | **Avg. add. effect**^2^ | **Avg. Dom. effect**^3^ | **Avg. α/σ_G_ in gen. 0**^4^ |  | **Total Nr.**^1^ | **Avg. add. effect**^2^ | **Avg. Dom. effect**^3^ | **Avg. α/σ_G_ in gen. 0**^4^ |
| ***Model A*** |  |  |  |  |  |  |  |  |  |  |  |  |  |  |  |
| **RANDOM** | 0.0 | 0.002 | - | 0.0001 |  | - | - | - | - | - |  | 1.8 | 0.017 | - | 0.0008 |
| **MASS** | 0.0 | 0.002 | - | 0.0001 |  | 0.9 | 0.010 | 0.101 | - | 0.0052 |  | 0.9 | 0.020 | - | 0.0010 |
| **PBLUP_OP** | 0.0 | 0.002 | - | 0.0001 |  | 1.3 | 0.006 | 0.036 | - | 0.0018 |  | 0.8 | 0.061 | - | 0.0031 |
| **GBLUP_NoOP** | 0.0 | 0.002 | - | 0.0001 |  | 1.3 | 0.013 | 0.045 | - | 0.0023 |  | 0.8 | 0.031 | - | 0.0016 |
| **GBLUP_OP** | 0.0 | 0.002 | - | 0.0001 |  | 1.6 | 0.009 | 0.030 | - | 0.0015 |  | 0.6 | 0.027 | - | 0.0014 |
|  |  |  |  |  |  |  |  |  |  |  |  |  |  |  |  |
| ***Model AD*** |  |  |  |  |  |  |  |  |  |  |  |  |  |  |  |
| **RANDOM** | 0.0 | 0.002 | 0.001 | 0.0001 |  | - | - | - | - | - |  | 2.4 | 0.018 | 0.009 | 0.0009 |
| **MASS** | 0.0 | 0.002 | 0.001 | 0.0001 |  | 3.6 | 0.000 | 0.100 | 0.059 | 0.0072 |  | 1.0 | 0.025 | 0.013 | 0.0012 |
| **PBLUP_OP** | 0.0 | 0.002 | 0.001 | 0.0001 |  | 1.3 | 0.008 | 0.038 | 0.026 | 0.0029 |  | 0.9 | 0.034 | 0.015 | 0.0018 |
| **GBLUP_NoOP** | 0.0 | 0.002 | 0.001 | 0.0001 |  | 1.1 | 0.011 | 0.030 | 0.016 | 0.0023 |  | 0.7 | 0.058 | 0.019 | 0.0026 |
| **GBLUP_OP** | 0.0 | 0.002 | 0.001 | 0.0001 |  | 1.0 | 0.006 | 0.028 | 0.020 | 0.0020 |  | 0.6 | 0.032 | 0.017 | 0.0018 |
|  |  |  |  |  |  |  |  |  |  |  |  |  |  |  |  |
| ***Model ADE*** |  |  |  |  |  |  |  |  |  |  |  |  |  |  |  |
| **RANDOM** | 0.0 | 0.002 | 0.001 | 0.0001 |  | - | - | - | - | - |  | 1.6 | 0.016 | 0.007 | 0.0009 |
| **MASS** | 0.0 | 0.002 | 0.001 | 0.0001 |  | 1.1 | 0.028 | 0.081 | 0.062 | 0.0238 |  | 1.3 | 0.023 | 0.011 | 0.0008 |
| **PBLUP_OP** | 0.0 | 0.002 | 0.001 | 0.0001 |  | 0.8 | 0.034 | 0.062 | 0.032 | 0.0084 |  | 0.9 | 0.040 | 0.014 | 0.0018 |
| **GBLUP_NoOP** | 0.0 | 0.002 | 0.001 | 0.0001 |  | 0.8 | 0.034 | 0.045 | 0.034 | 0.0062 |  | 0.8 | 0.045 | 0.017 | 0.0022 |
| **GBLUP_OP** | 0.0 | 0.002 | 0.001 | 0.0001 |  | 0.8 | 0.028 | 0.068 | 0.027 | 0.0082 |  | 0.9 | 0.060 | 0.016 | 0.0018 |

^1^ Total number of loci.

^2^ Average absolute functional additive effect of loci that became fixed in the population.

^3^ Average absolute functional dominance effect of loci that became fixed in the population.

^4^ Average absolute statistical additive effect (α) divided by the total genetic standard deviation in generation 0 of loci that became fixed in the population.

**TABLE S4.3 –** Average number of interactions per locus for all loci, and loci with a large (>0.9) or small (<0.01) change in allele frequency across 50 generations of selection for the five selection methods and the genetic model with additive, dominance and epistatic effect^1^. The five selection methods were: RANDOM selection, MASS selection, PBLUP selection with own performance (PBLUP_OP), GBLUP selection without own performance (GBLUP_NoOP) or with own performance (GBLUP_OP).

|  | **Average number of interactions of loci** | | | | | |
| --- | --- | --- | --- | --- | --- | --- |
|  | **All loci** | | **Loci with large (>0.9) change in allele frequency** | | **Loci with small (<0.01) change in allele frequency** | |
| **RANDOM** | 121 | (0.4) | - | - | 85 | (7.3) |
| **MASS** | 121 | (0.4) | 459 | (60.1) | 105 | (5.4) |
| **PBLUP_OP** | 121 | (0.4) | 234 | (20.1) | 85 | (7.3) |
| **GBLUP_NoOP** | 121 | (0.4) | 193 | (16.2) | 113 | (9.9) |
| **GBLUP_OP** | 121 | (0.4) | 243 | (18.5) | 93 | (8.5) |

^1^ Results are shown as averages across the loci over all replicates with their corresponding standard errors of the mean between brackets.
