# Supplementary material for: The long-term effects of genomic selection: 2. Changes in allele frequencies of causal loci and new mutations
Source: Genetics. 2023 Jul 28;225(1):iyad141. doi: 10.1093/genetics/iyad141 (PMC10471209; doi:10.1093/genetics/iyad141)
Supplement: iyad141_Supplementary_Data [file iyad141_supplementary_data.zip › File_S5_GENETICS-2023-306366.docx]

**File S5: Apparent effect versus statistical additive effects**


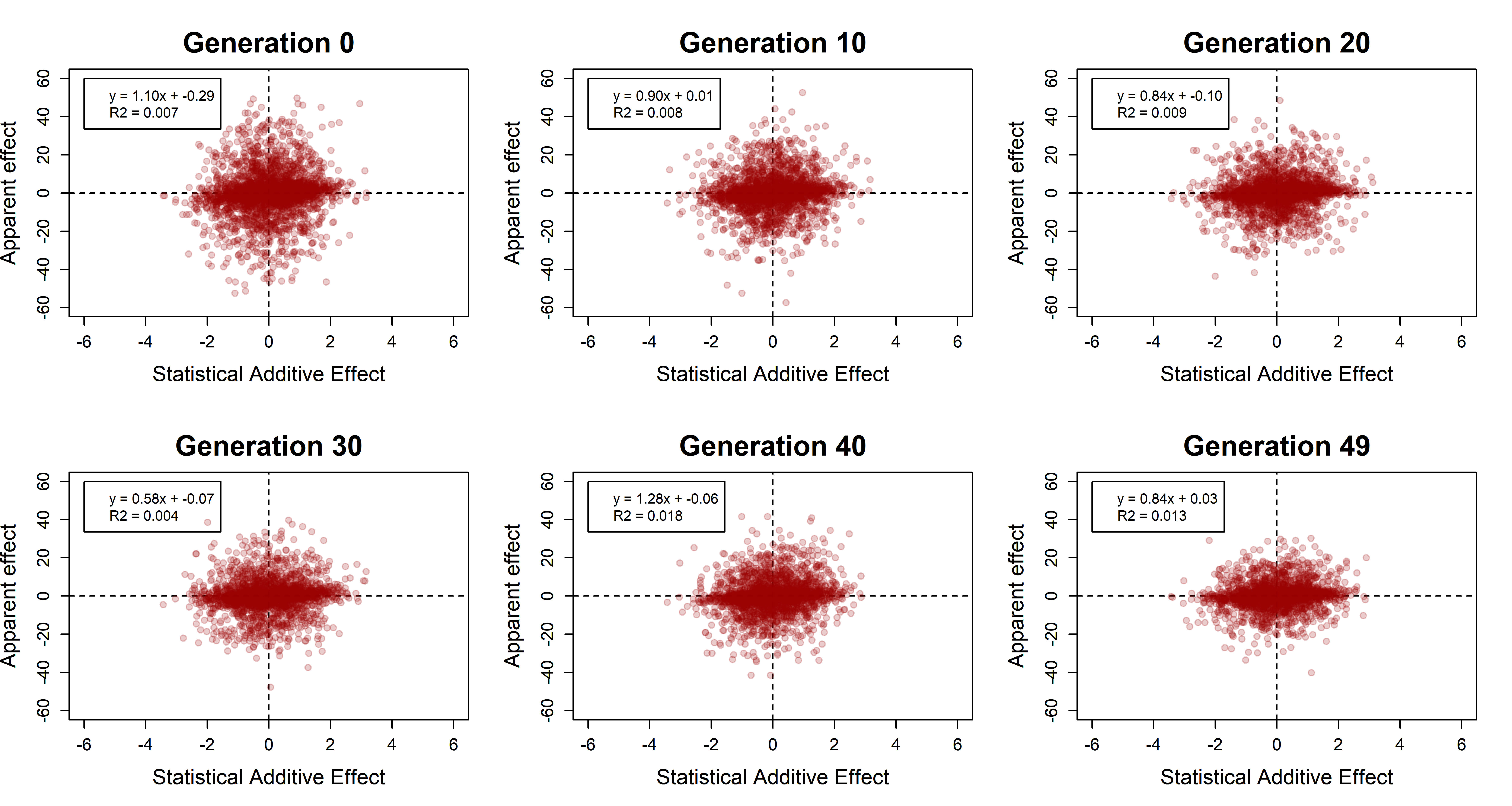
**FIGURE S5.1**

Apparent effects versus statistical additive effects across all segregating causal loci in different generations for the genetic model with additive effects (Model A) under MASS selection.


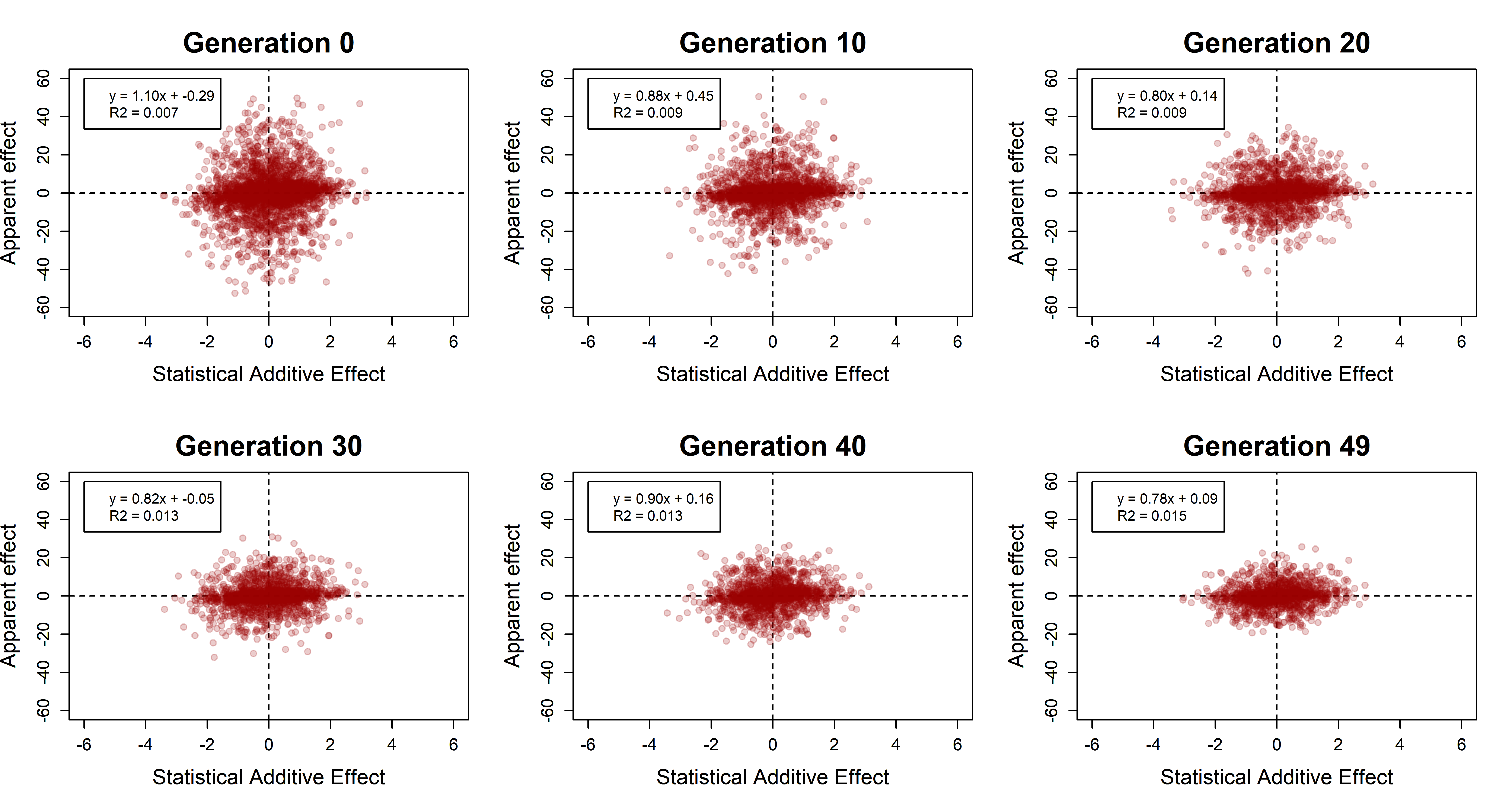
**FIGURE S5.2**

Apparent effects versus statistical additive effects across all segregating causal loci in different generations for the genetic model with additive effects (Model A) under PBLUP selection with own performance (PBLUP_OP).


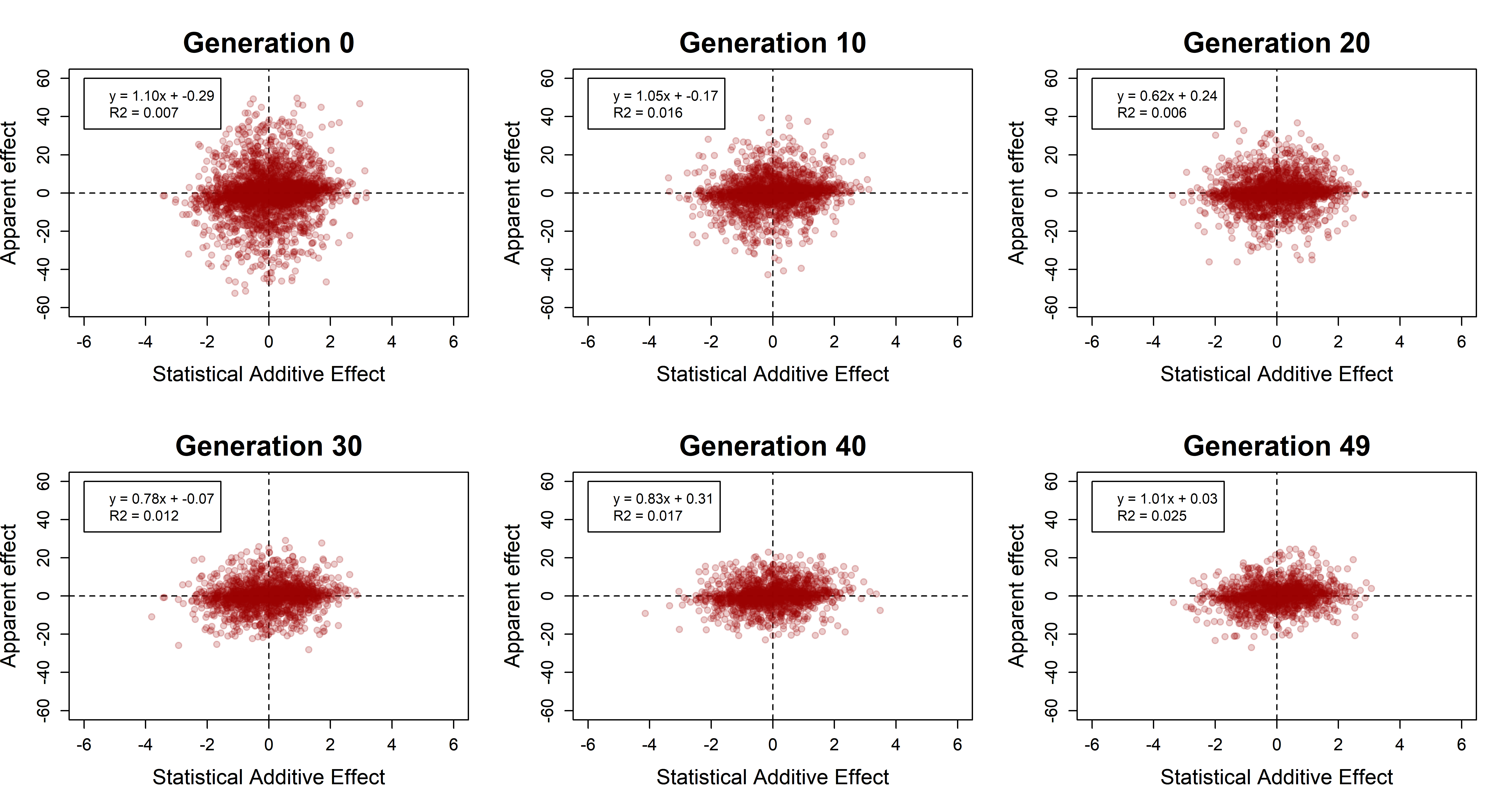
**FIGURE S5.3**

Apparent effects versus statistical additive effects across all segregating causal loci in different generations for the genetic model with additive effects (Model A) under GBLUP selection without own performance (GBLUP_NoOP).


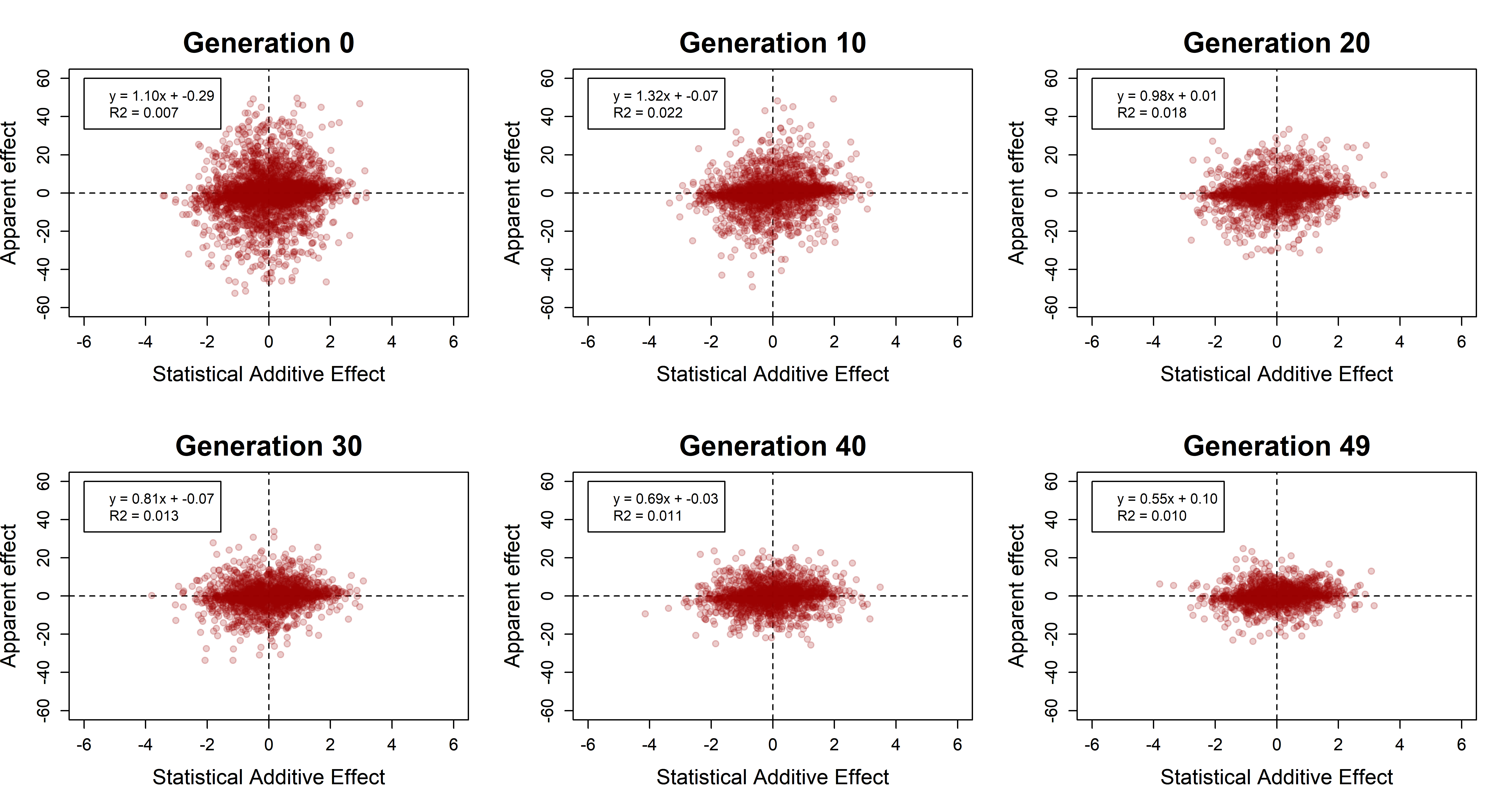
**FIGURE S5.4**

Apparent effects versus statistical additive effects across all segregating causal loci in different generations for the genetic model with additive effects (Model A) under GBLUP selection with own performance (GBLUP_OP).


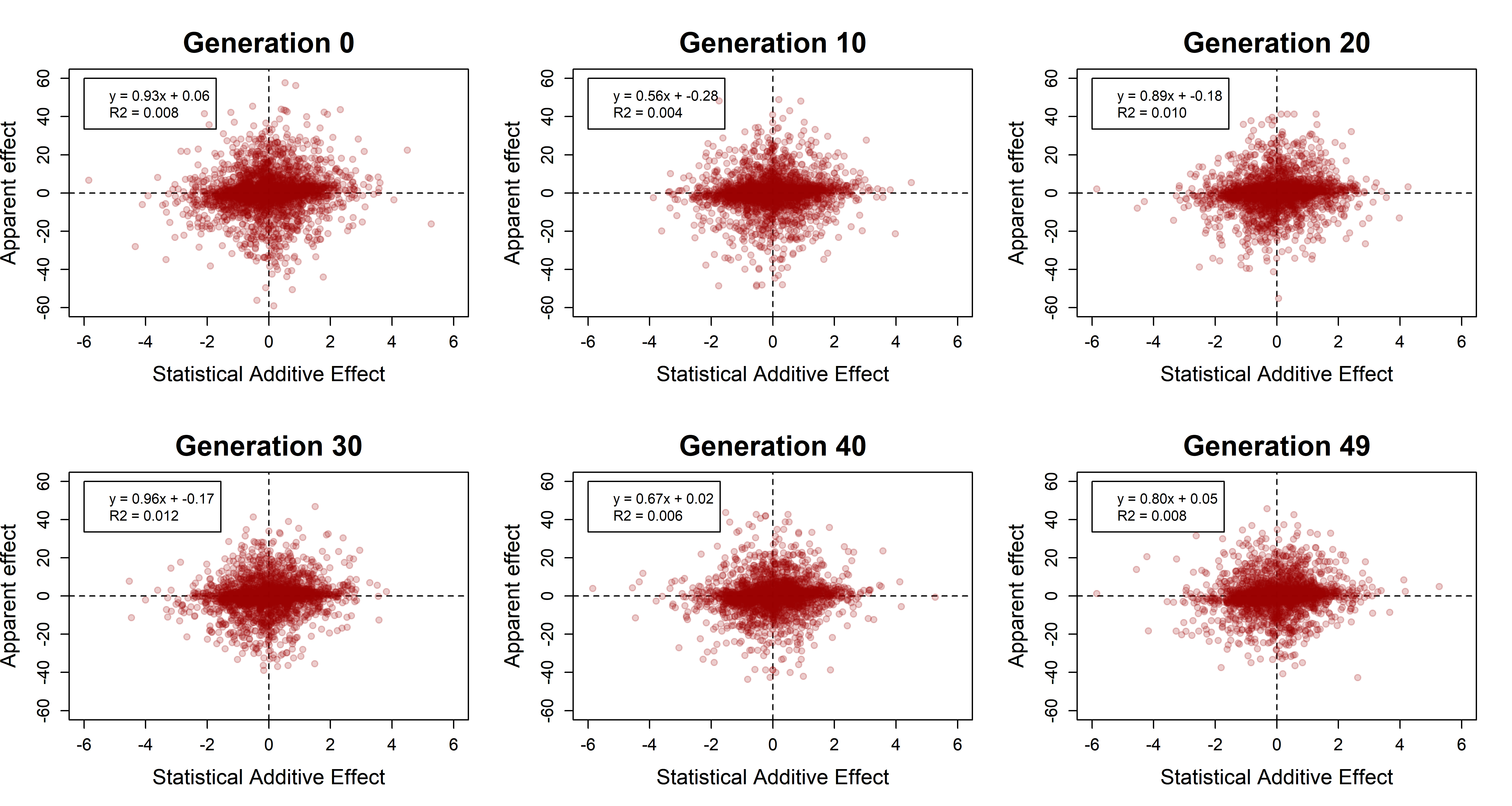
**FIGURE S5.5**

Apparent effects versus statistical additive effects across all segregating causal loci in different generations for the genetic model with additive and dominance effects (Model AD) under MASS selection.


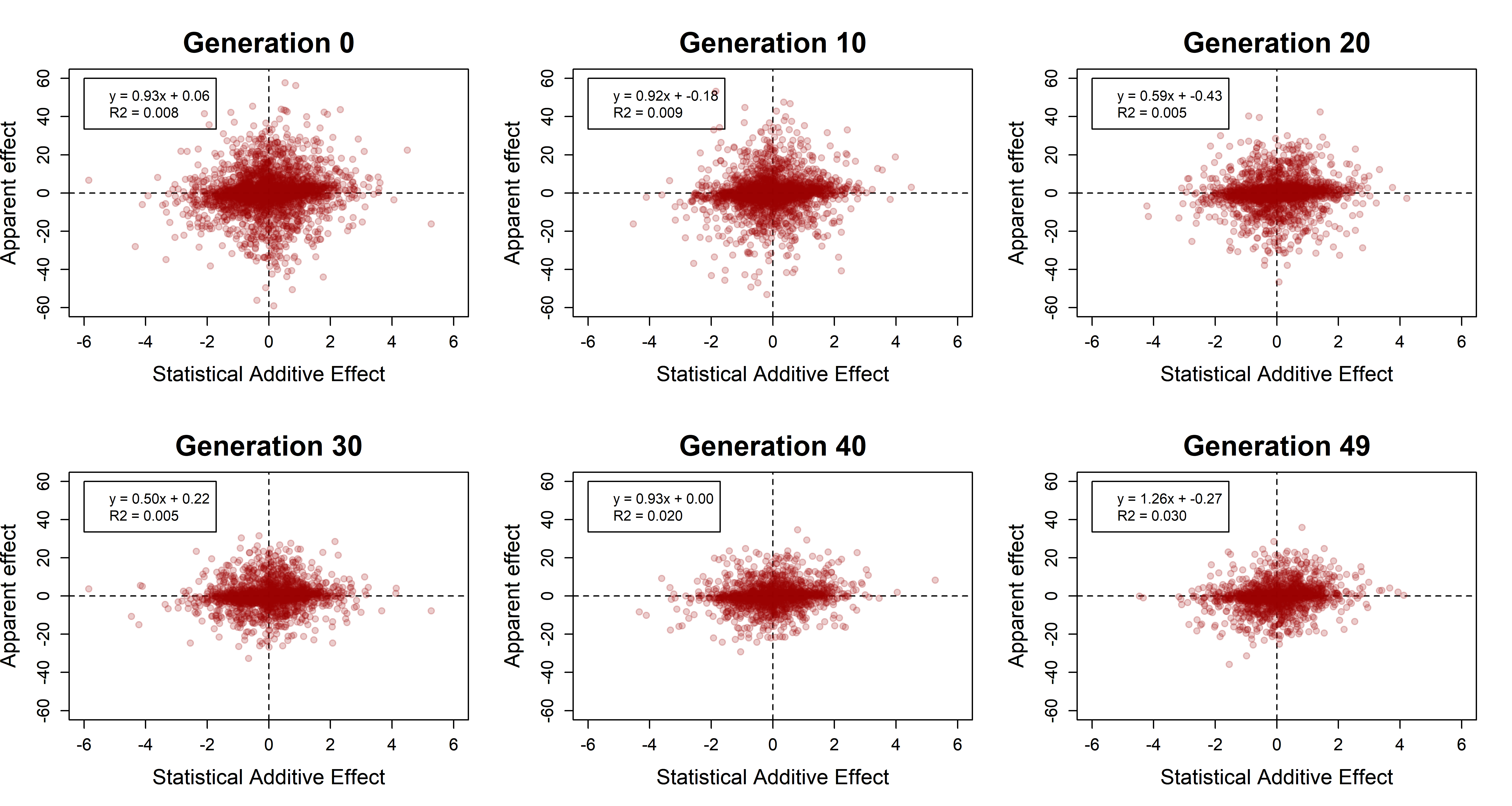
**FIGURE S5.6**

Apparent effects versus statistical additive effects across all segregating causal loci in different generations for the genetic model with additive and dominance effects (Model AD) under PBLUP selection with own performance (PBLUP_OP).


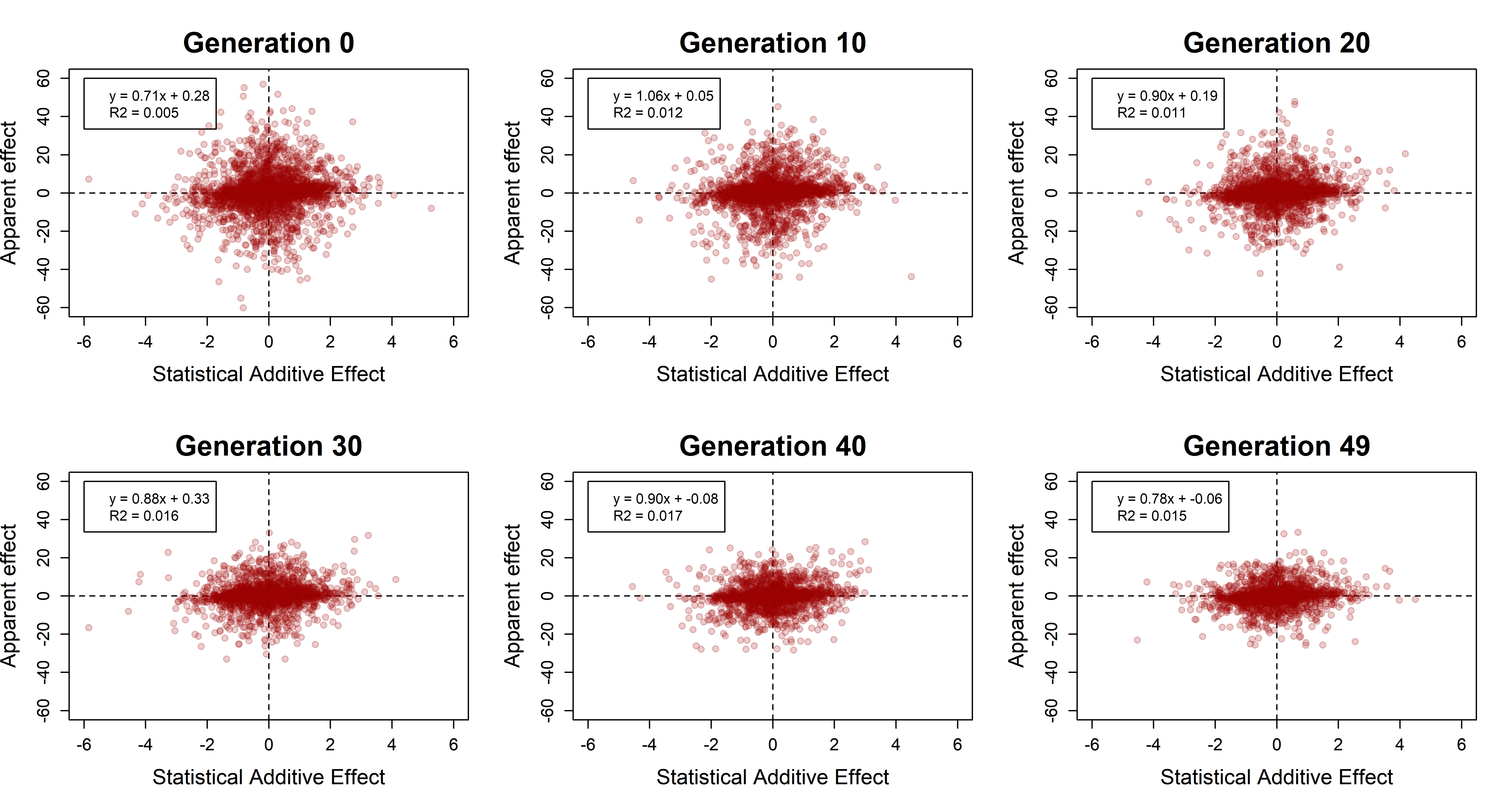
**FIGURE S5.7**

Apparent effects versus statistical additive effects across all segregating causal loci in different generations for the genetic model with additive and dominance effects (Model AD) under GBLUP selection without own performance (GBLUP_NoOP).


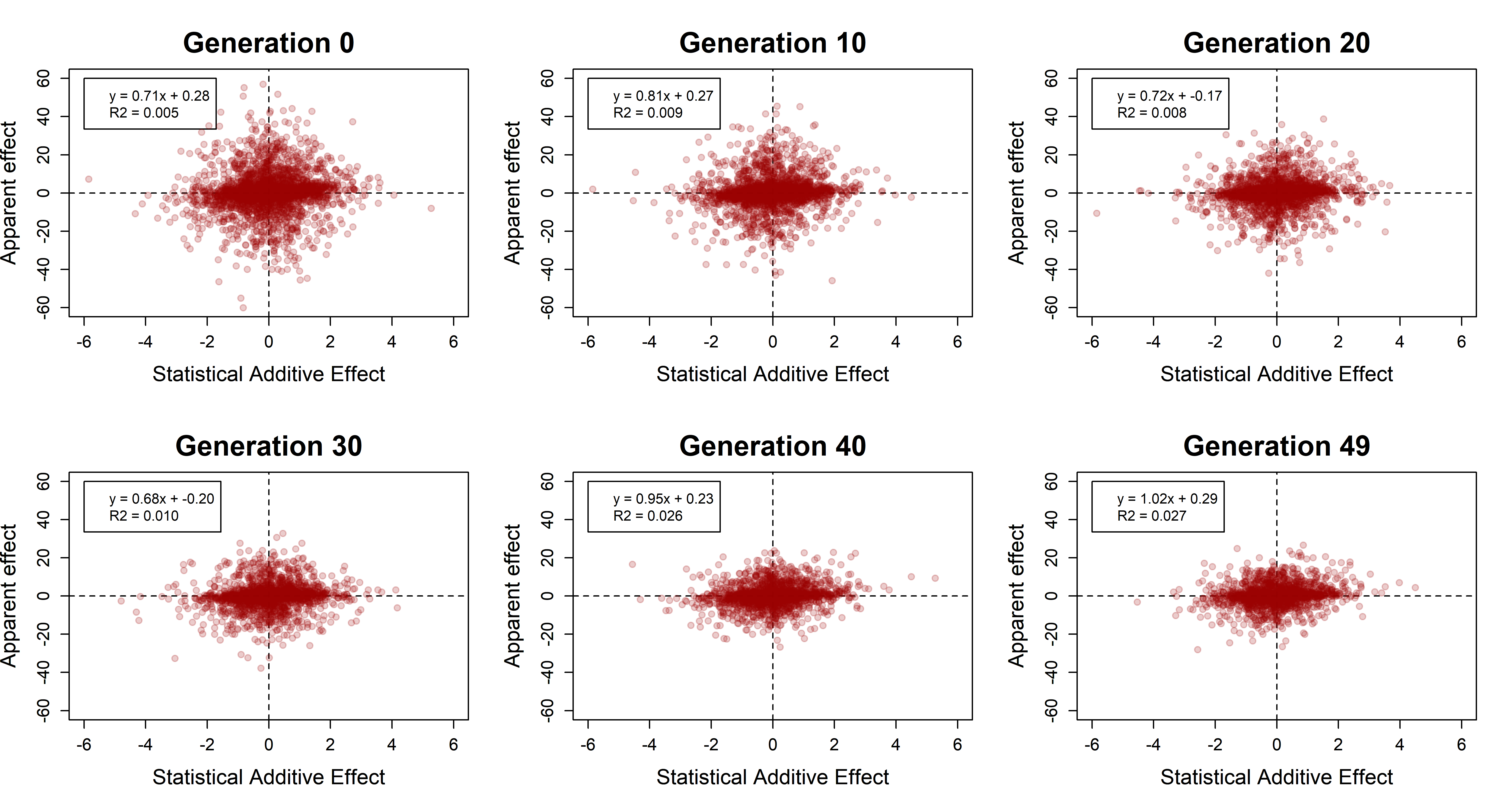
**FIGURE S5.8**

Apparent effects versus statistical additive effects across all segregating causal loci in different generations for the genetic model with additive and dominance effects (Model AD) under GBLUP selection with own performance (GBLUP_OP).


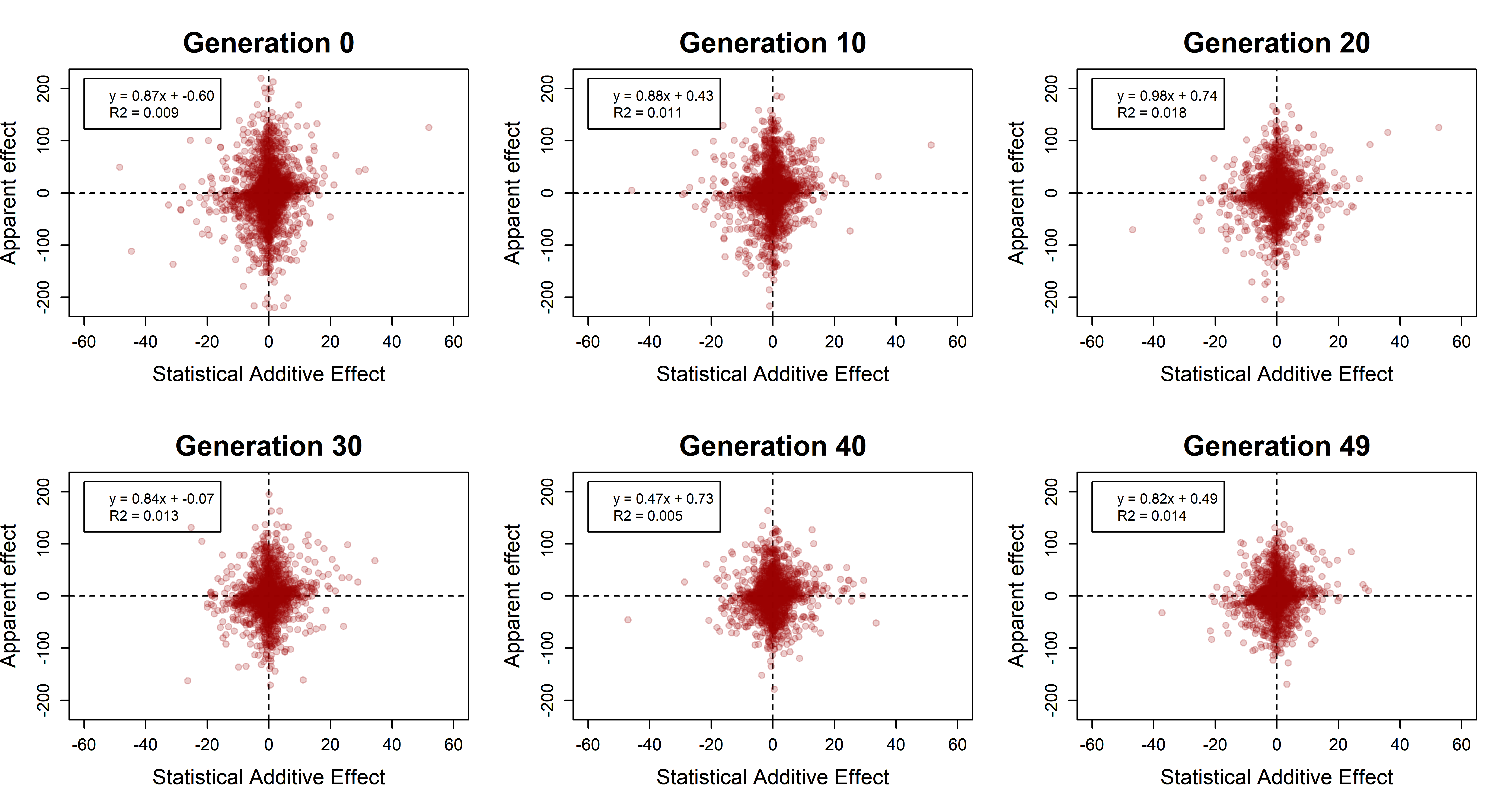
**FIGURE S5.9**

Apparent effects versus statistical additive effects across all segregating causal loci in different generations for the genetic model with additive, dominance and epistatic effects (Model ADE) under MASS selection.


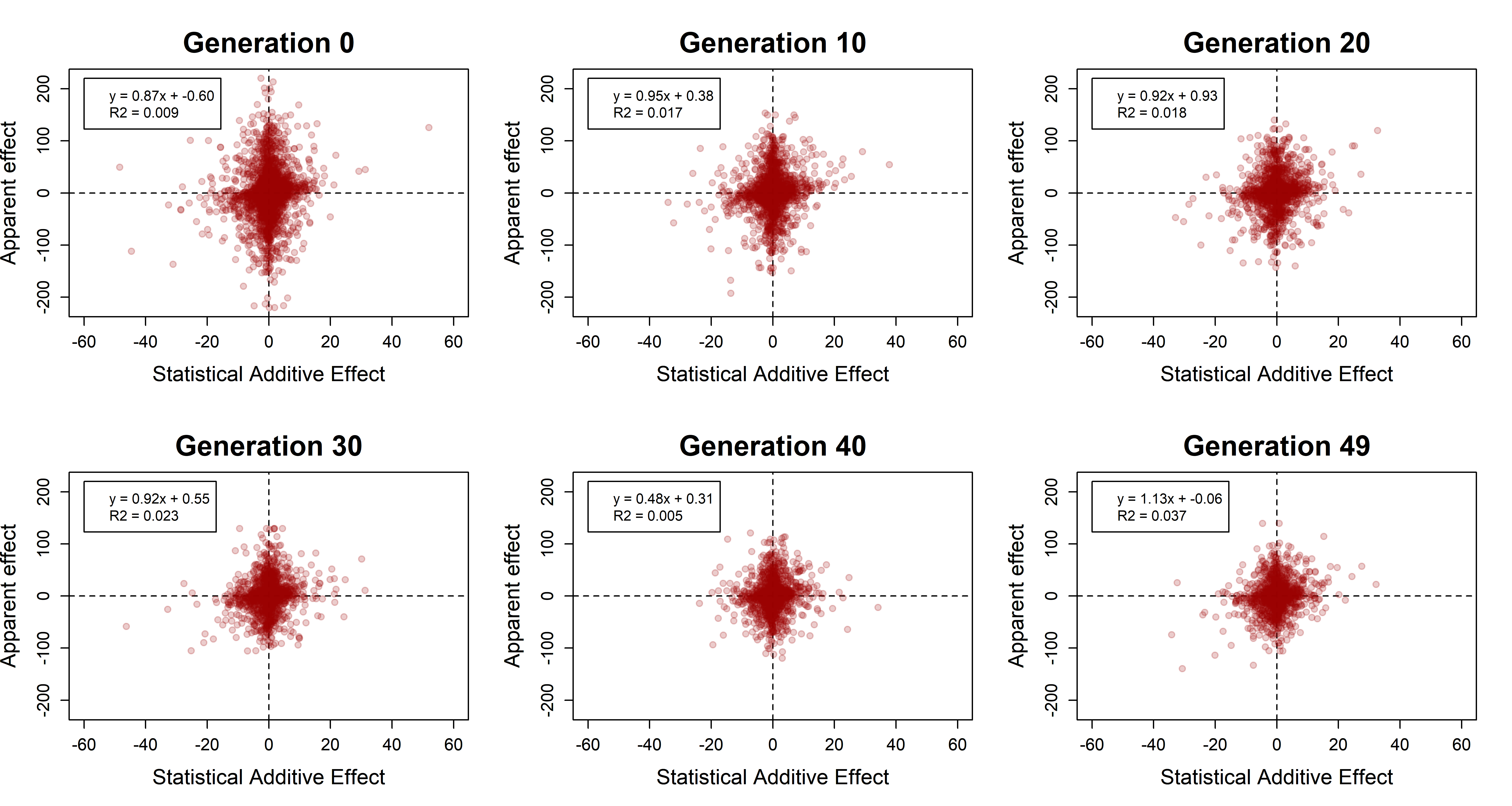
**FIGURE S5.10**

Apparent effects versus statistical additive effects across all segregating causal loci in different generations for the genetic model with additive, dominance and epistatic effects (Model ADE) under PBLUP selection with own performance (PBLUP_OP).


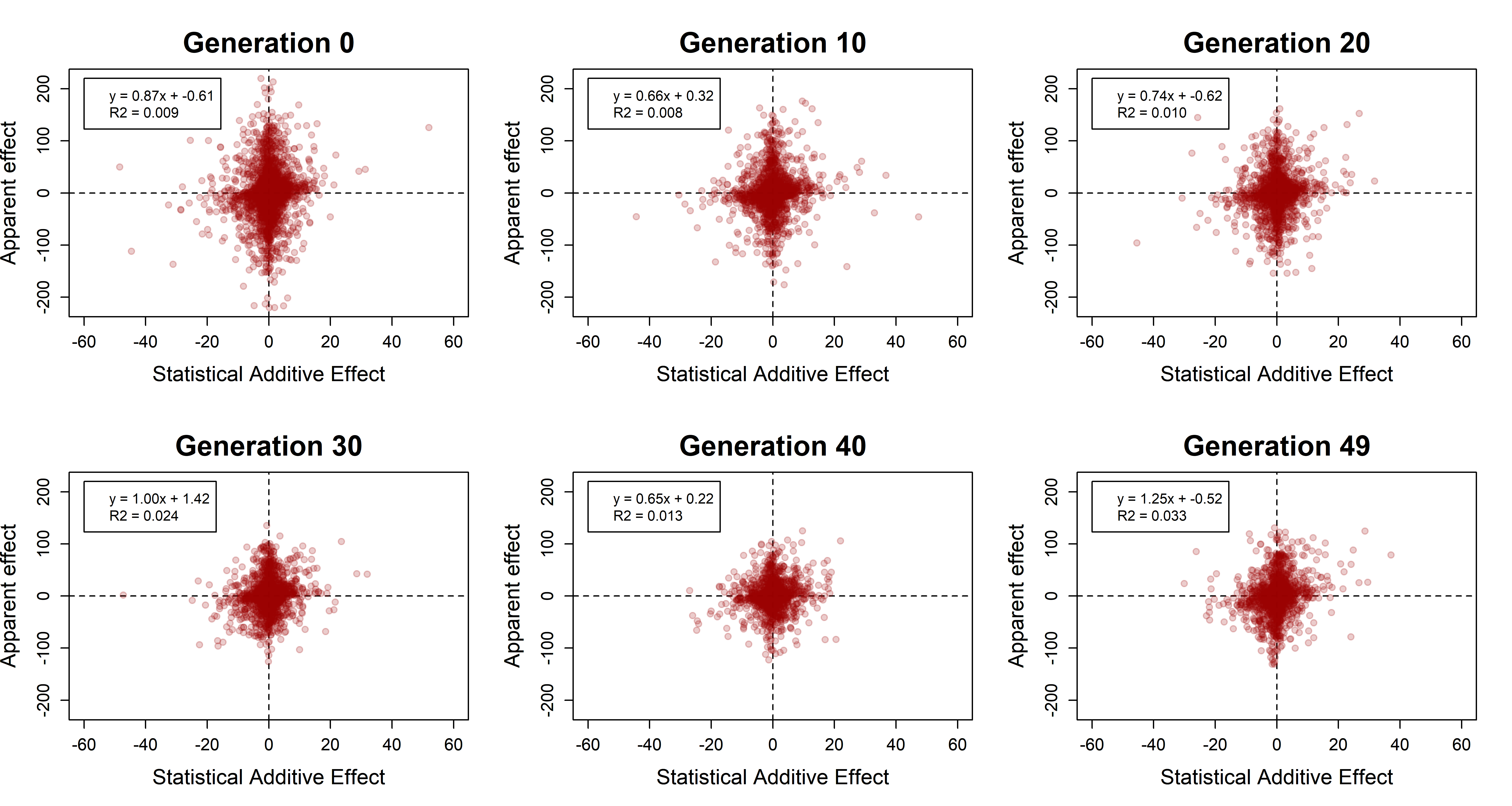
**FIGURE S5.11**

Apparent effects versus statistical additive effects across all segregating causal loci in different generations for the genetic model with additive, dominance and epistatic effects (Model ADE) under GBLUP selection without own performance (GBLUP_NoOP).


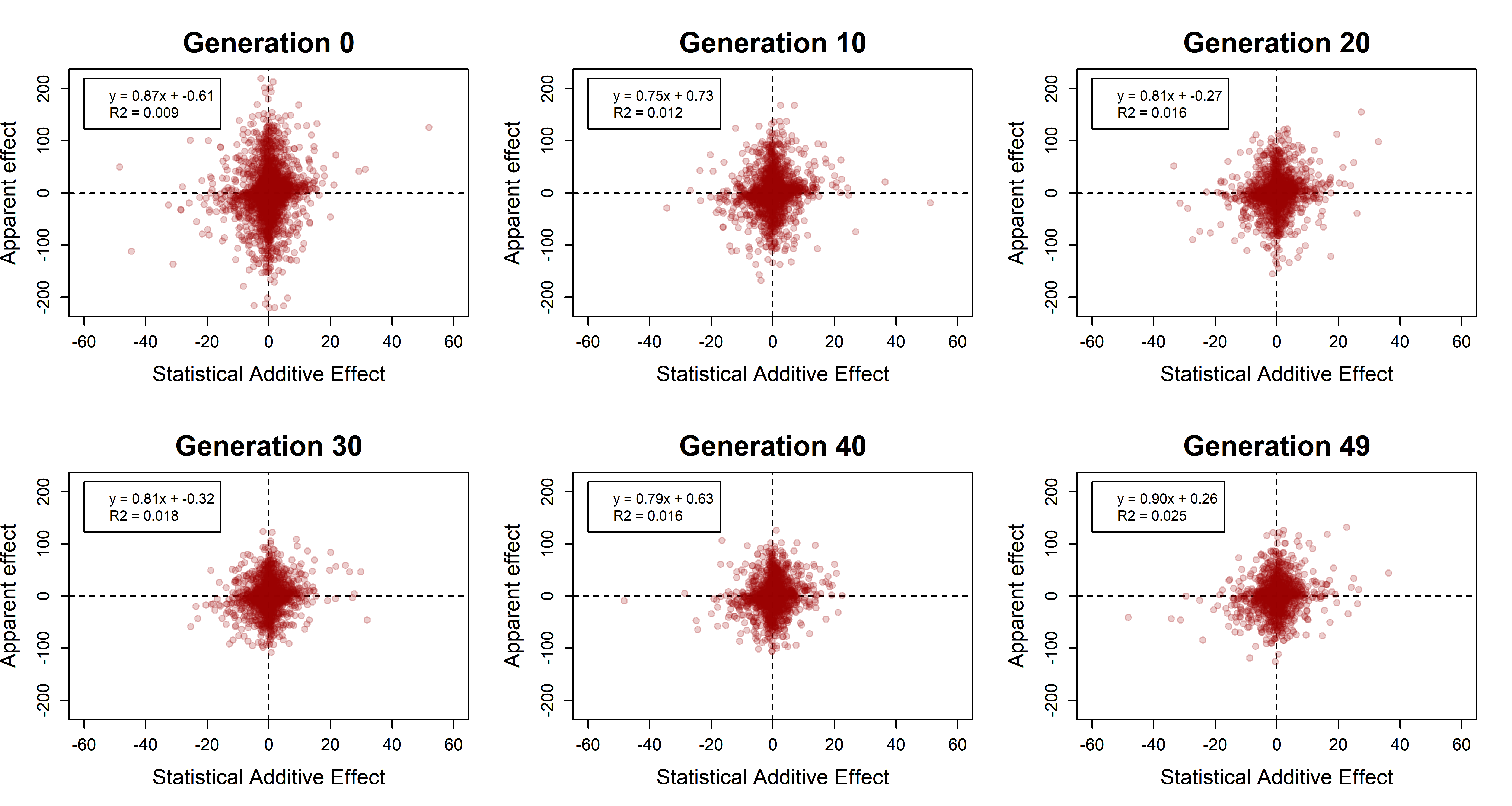
**FIGURE S5.12**

Apparent effects versus statistical additive effects across all segregating causal loci in different generations for the genetic model with additive, dominance and epistatic effects (Model ADE) under GBLUP selection with own performance (GBLUP_OP).
