# Supplementary material for: The long-term effects of genomic selection: 2. Changes in allele frequencies of causal loci and new mutations
Source: Genetics. 2023 Jul 28;225(1):iyad141. doi: 10.1093/genetics/iyad141 (PMC10471209; doi:10.1093/genetics/iyad141)
Supplement: iyad141_Supplementary_Data [file iyad141_supplementary_data.zip › File_S1_GENETICS-2023-306366.docx]

**File S1: Supplementary figures**


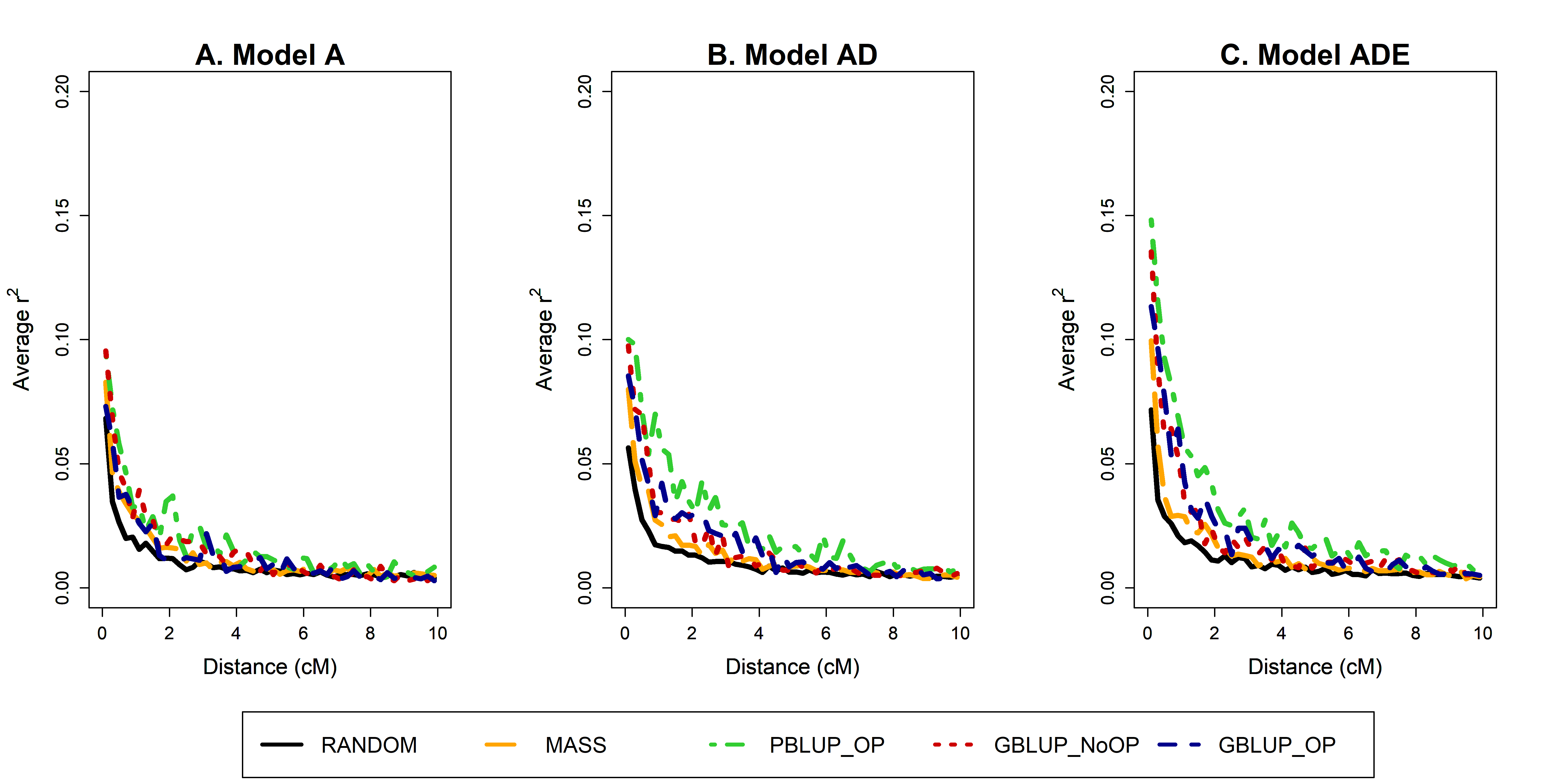


**FIGURE S1.1**

Linkage disequilibrium pattern between causal loci (expressed as *r^2^*) after 50 generations of selection for the five selection methods and three genetic models. The five selection methods were: RANDOM selection, MASS selection, PBLUP selection with own performance (PBLUP_OP), GBLUP selection without own performance (GBLUP_NoOP) or with own performance (GBLUP_OP). The three genetic models were a model with only additive effects (A), with additive and dominance effects (AD), or with additive, dominance and epistatic effects (ADE). Results are shown as averages of 20 replicates.


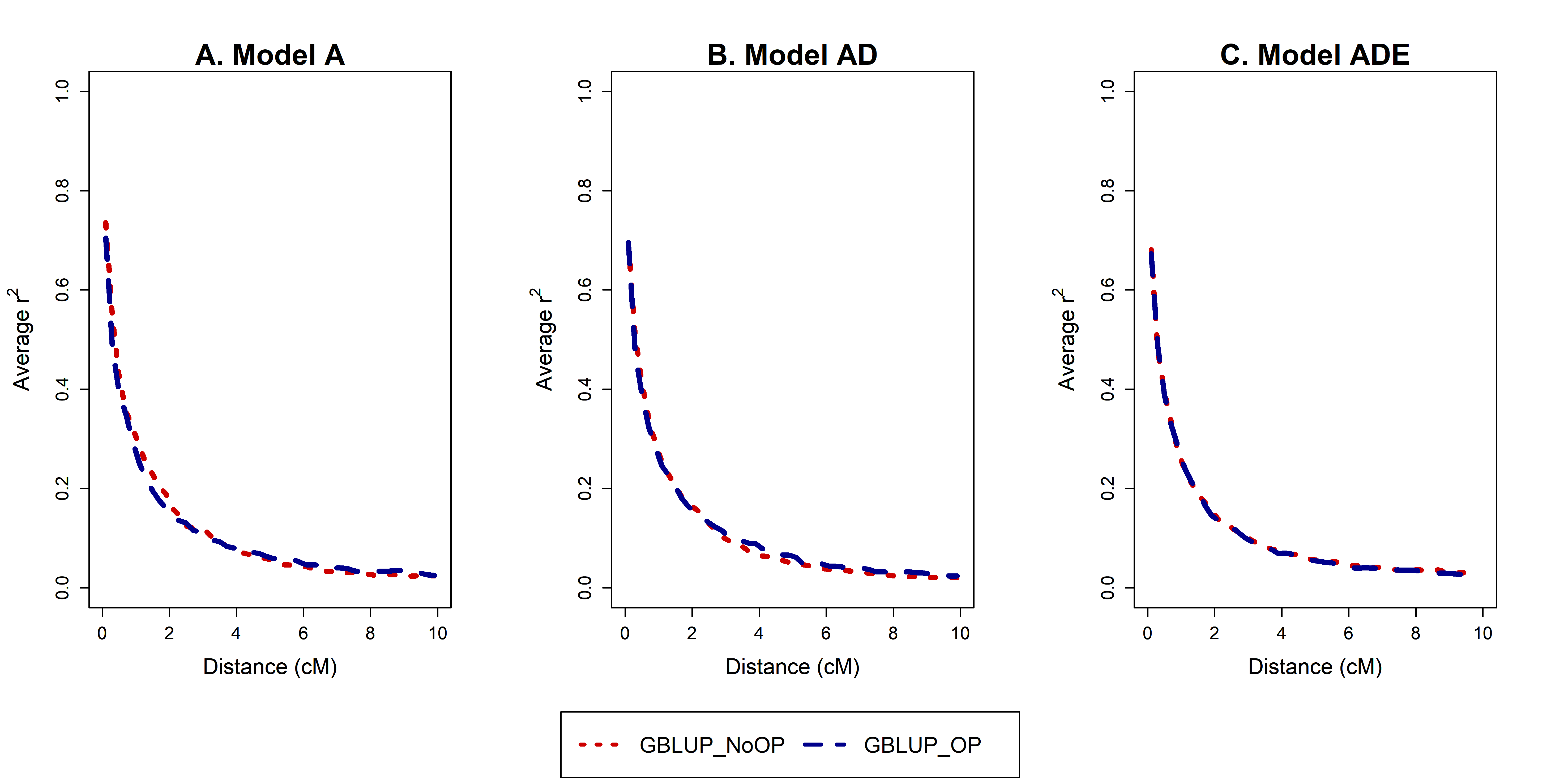


**FIGURE S1.2**

Linkage disequilibrium pattern between markers (expressed as *r^2^*) after 50 generations of selection for two selection methods and three genetic models. The two selection methods were: GBLUP selection without own performance (GBLUP_NoOP) or with own performance (GBLUP_OP). No markers were simulated for the other selection methods. The three genetic models were a model with only additive effects (A), with additive and dominance effects (AD), or with additive, dominance and epistatic effects (ADE). Results are shown as averages of 20 replicates.


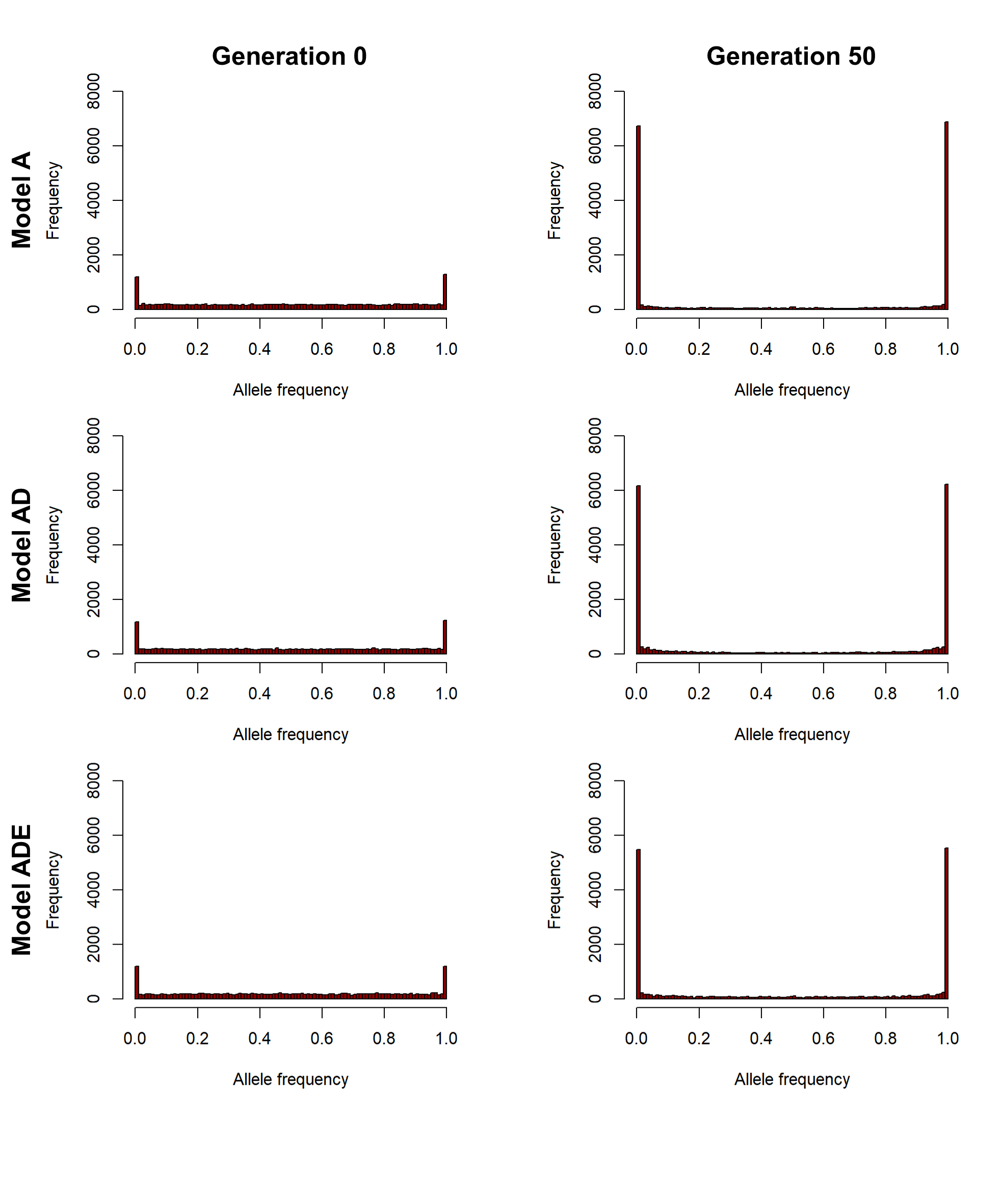


**FIGURE S1.3**

Allele frequency distribution of marker before (Generation 0) and after (Generation 50) selection. Results are given for one replicate for GBLUP selection with own performance (GBLUP_OP) for three genetic models: model A with only additive effects, model AD with additive and dominance effects and model ADE with additive, dominance and epistatic effects.


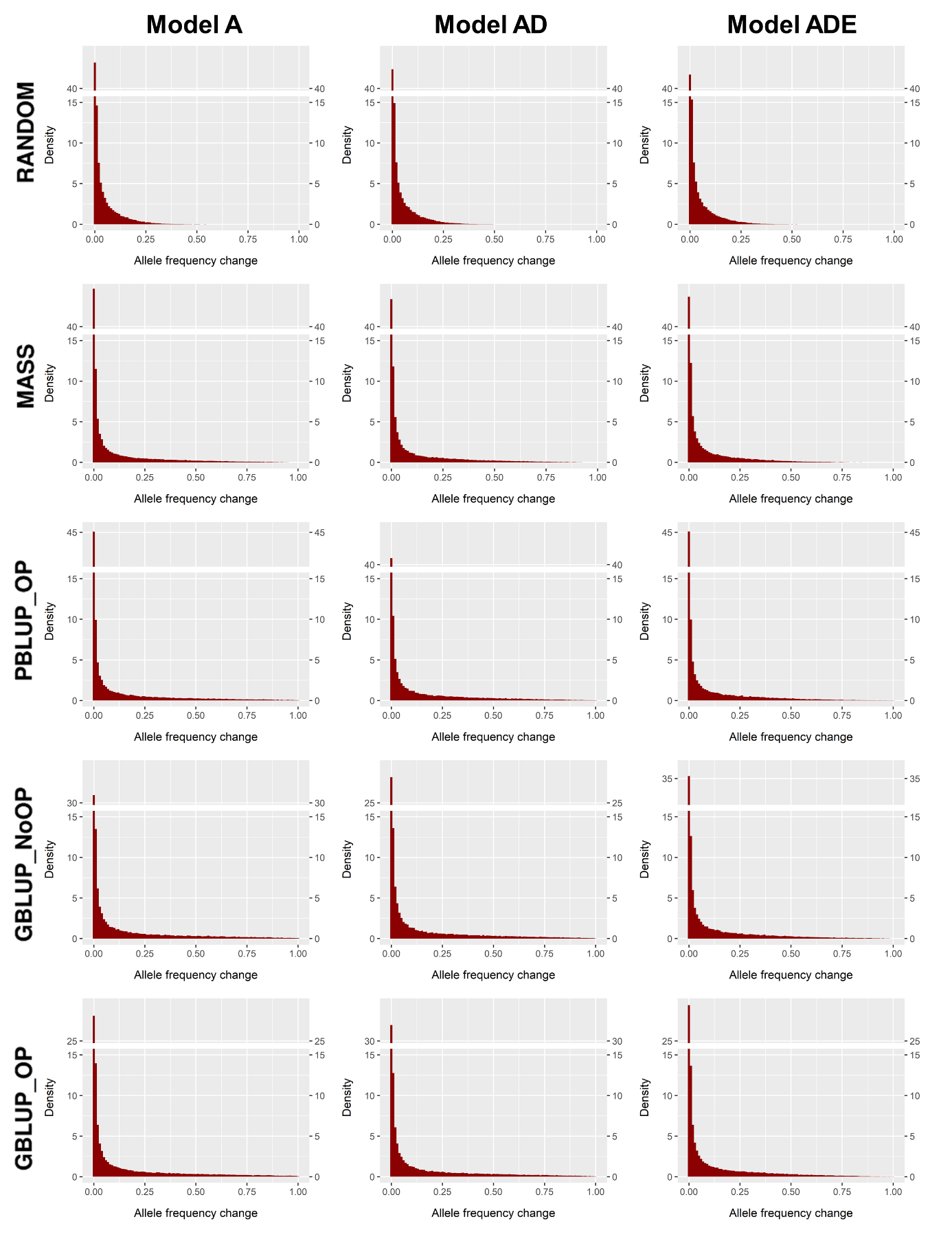


**FIGURE S1.4**

Distribution in absolute change in allele frequency of causal loci over 50 generations of selection for five selection methods and three genetic models. The five selection methods were: RANDOM selection, MASS selection, PBLUP selection with own performance (PBLUP_OP), GBLUP selection without own performance (GBLUP_NoOP) or with own performance (GBLUP_OP). The three genetic models were a model with only additive effects (A), with additive and dominance effects (AD), or with additive, dominance and epistatic effects (ADE). Results are cumulative across the 20 replicates.


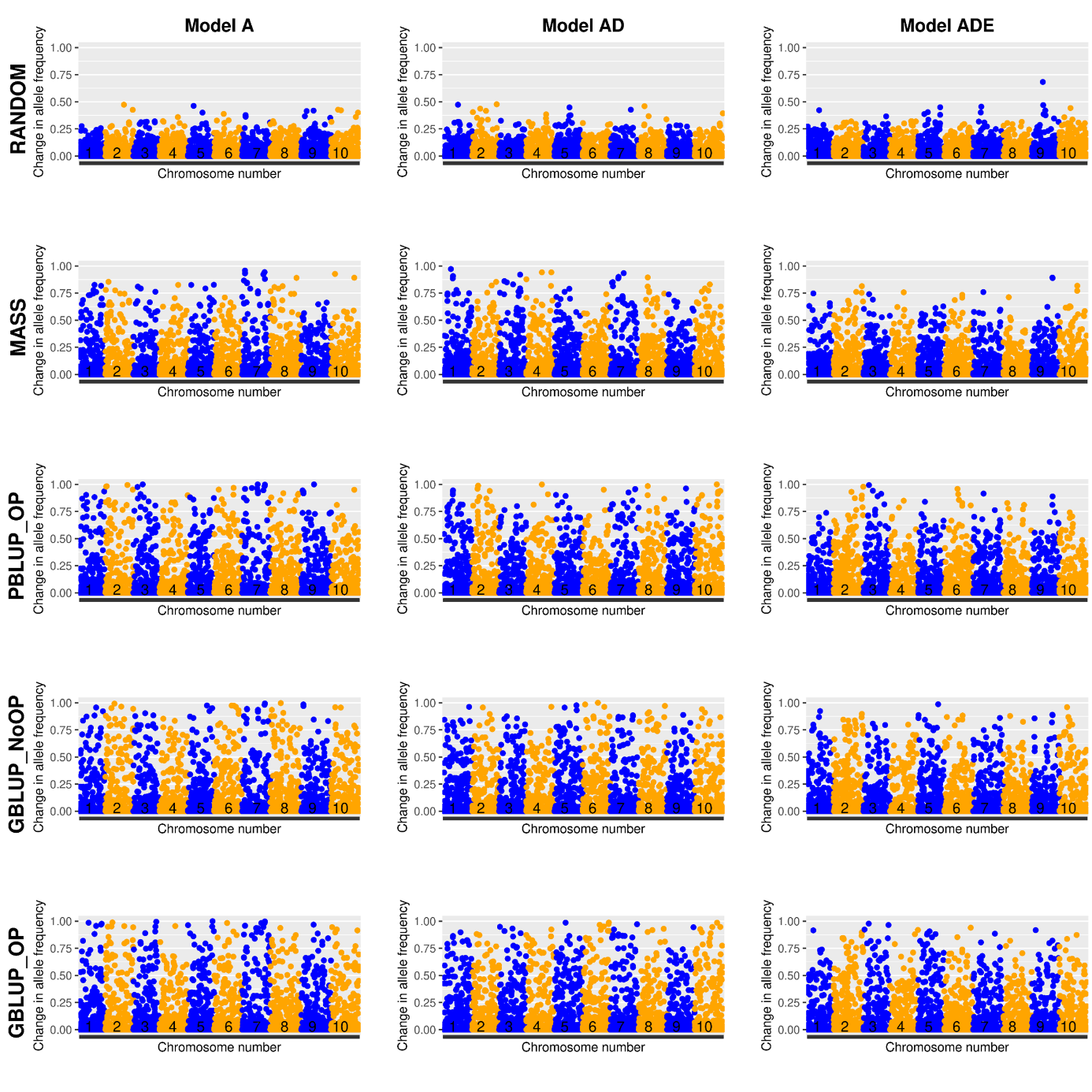


**FIGURE S1.5**

Absolute change in allele frequency of causal loci over 50 generations of selection versus the position on the genome for the five selection methods and three genetic models. The five selection methods were: RANDOM selection, MASS selection, PBLUP selection with own performance (PBLUP_OP), GBLUP selection without own performance (GBLUP_NoOP) or with own performance (GBLUP_OP). The three genetic models were a model with only additive effects (A), with additive and dominance effects (AD), or with additive, dominance and epistatic effects (ADE). Results are shown for one replicate.


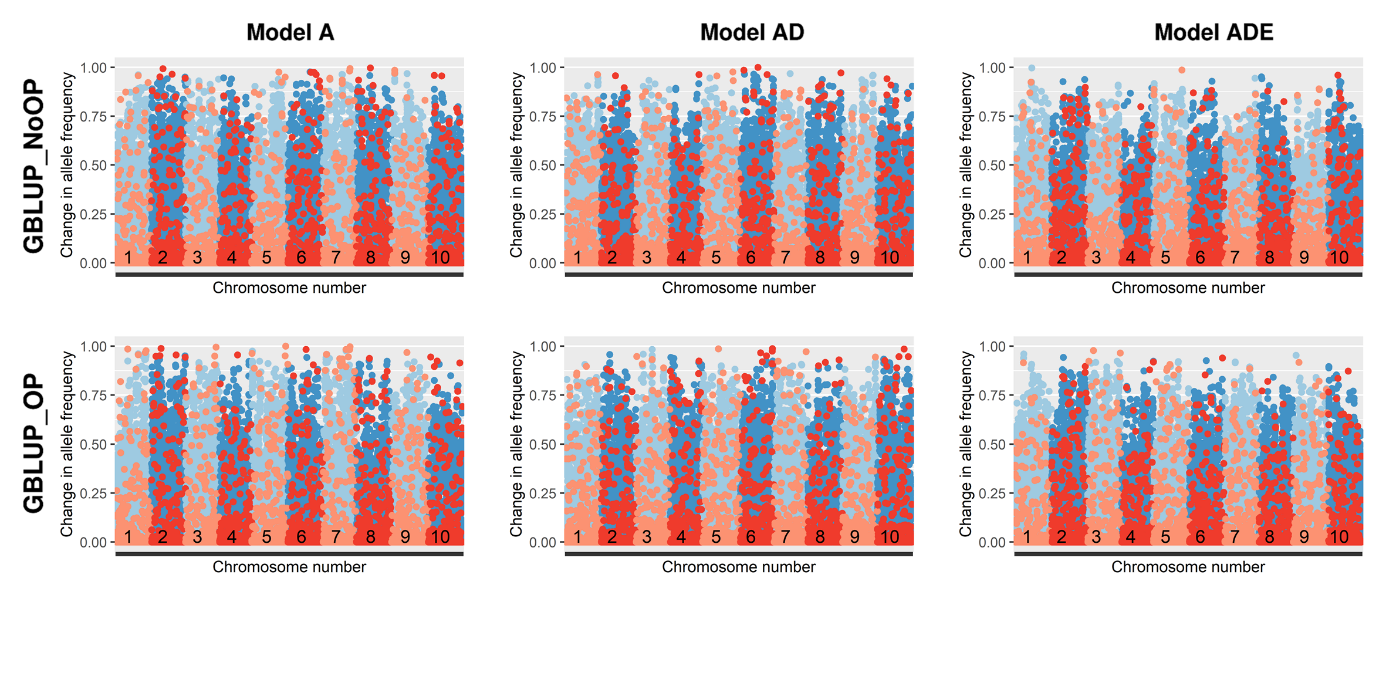


**FIGURE S1.6**

Absolute change in allele frequency of causal loci (red colours) and markers (blue colours) over 50 generations of selection versus the position of the genome for two selection methods and three genetic models. The two selection methods were: GBLUP selection without own performance (GBLUP_NoOP) or with own performance (GBLUP_OP). The three genetic models were a model with only additive effects (A), with additive and dominance effects (AD), or with additive, dominance and epistatic effects (ADE). Results are shown for one replicate.


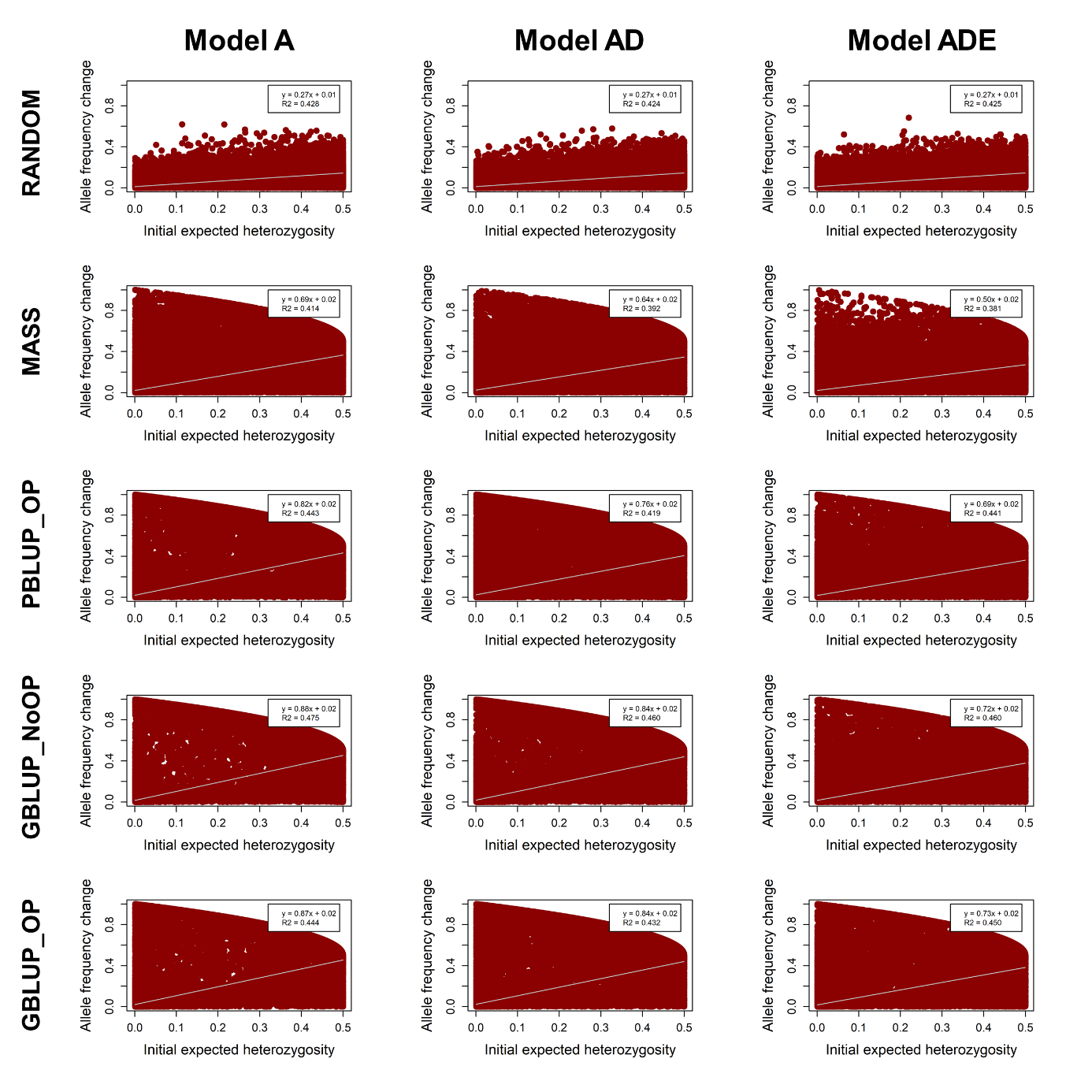


**FIGURE S1.7**

Absolute change in allele frequency of causal loci over 50 generations of selection versus the initial expected heterozygosity for five selection methods and three genetic models. The initial expected heterozygosity was calculated as $2p_{i}\left( 1-p_{i} \right)$, where *p_i_* is the allele frequency of locus *i* in generation 0. The five selection methods were: RANDOM selection, MASS selection, PBLUP selection with own performance (PBLUP_OP), GBLUP selection without own performance (GBLUP_NoOP) or with own performance (GBLUP_OP). The three genetic models were a model with only additive effects (A), with additive and dominance effects (AD), or with additive, dominance and epistatic effects (ADE). Results are cumulative across the 20 replicates.


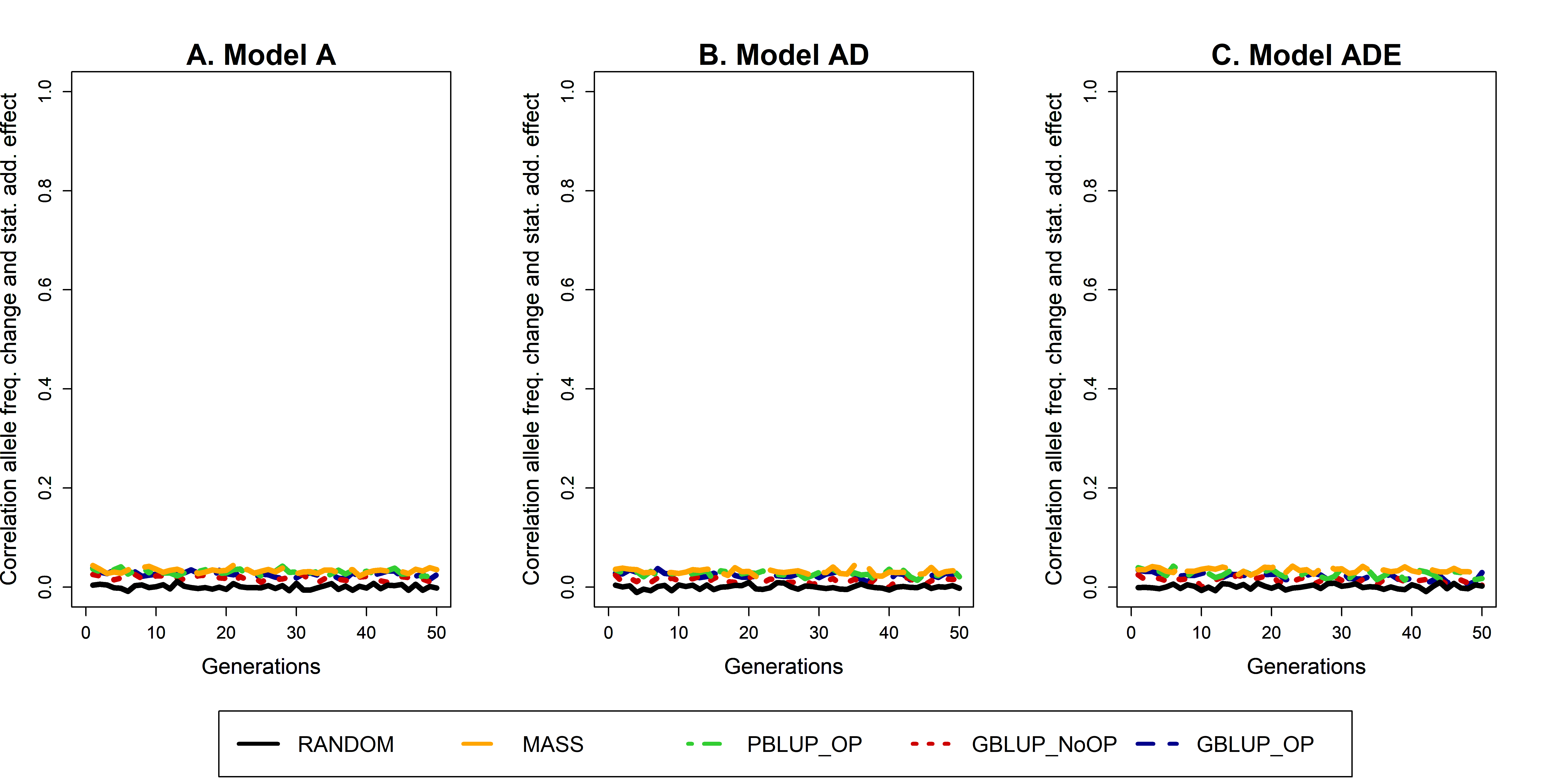
**FIGURE S1.8**

Correlation between the change in allele frequency towards the next generations and the statistical additive effect for the five selection methods and three genetic models. The change in allele frequency is expressed as the change in allele frequency from generation *i* to generation *i* +1 divided by *p_i_*(1-*p_i_*), where *p_i_* is the allele frequency in generation *i*. The five selection methods were: RANDOM selection, MASS selection, PBLUP selection with own performance (PBLUP_OP), GBLUP selection without own performance (GBLUP_NoOP) or with own performance (GBLUP_OP). The three genetic models were a model with only additive effects (A), with additive and dominance effects (AD), or with additive, dominance and epistatic effects (ADE). Results are shown as averages of 20 replicates.


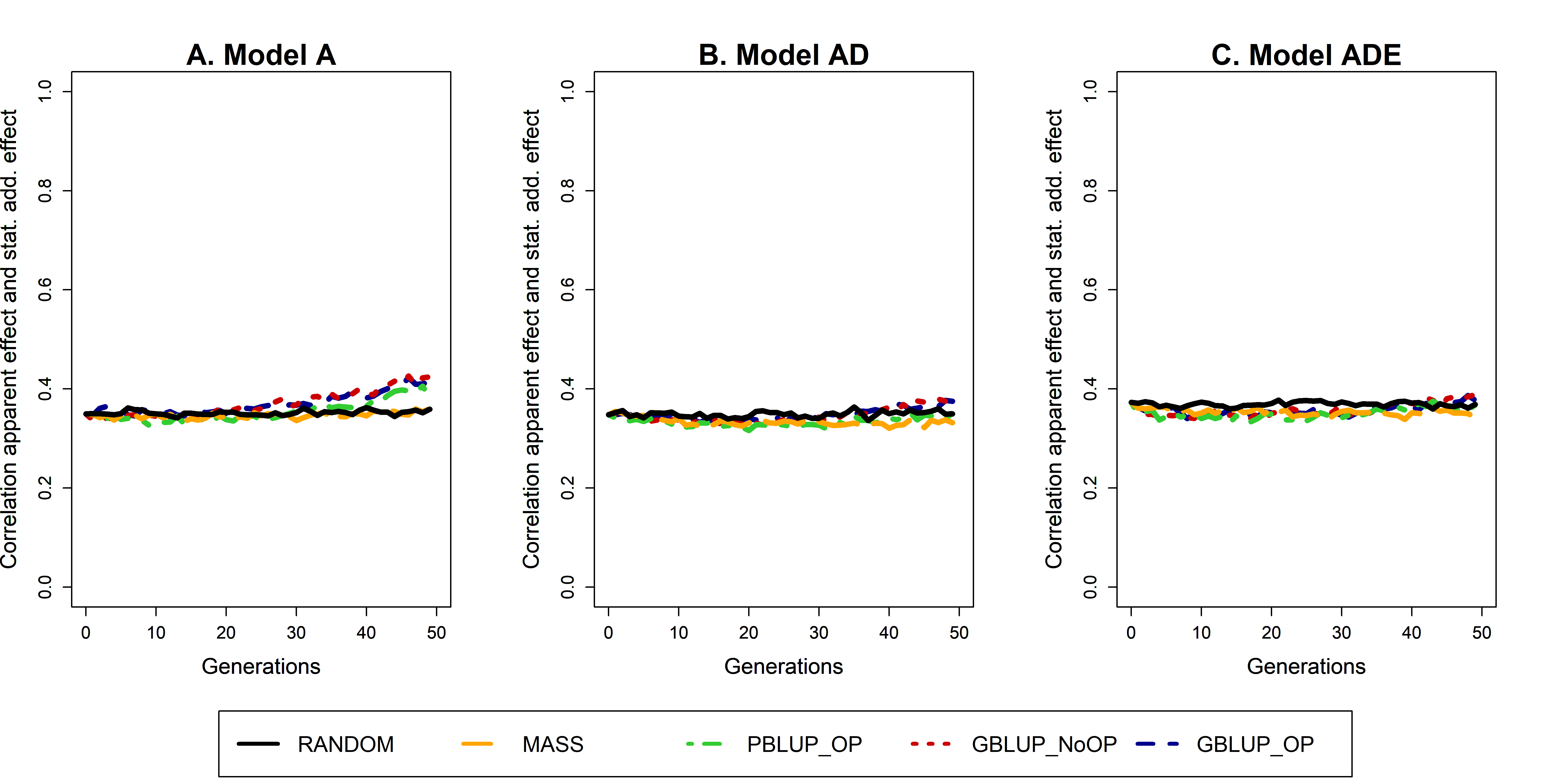


**FIGURE S1.9**

Correlation between the apparent effect and the statistical additive effect across generations for loci with a minor allele frequency above 0.05 for the five selection methods and three genetic models. The five selection methods were: RANDOM selection, MASS selection, PBLUP selection with own performance (PBLUP_OP), GBLUP selection without own performance (GBLUP_NoOP) or with own performance (GBLUP_OP). The three genetic models were a model with only additive effects (A), with additive and dominance effects (AD), or with additive, dominance and epistatic effects (ADE). Results are shown as averages of 20 replicates.


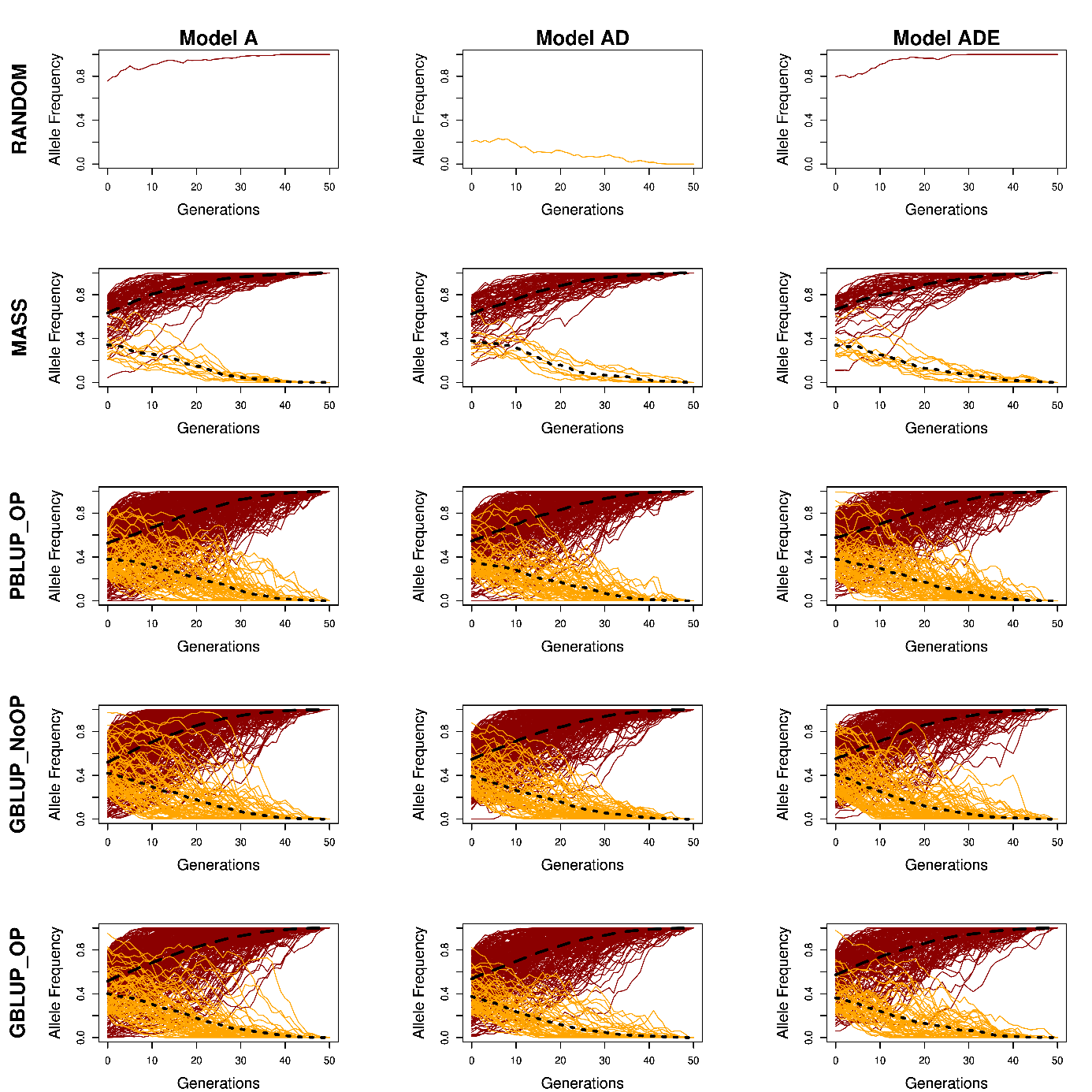


**FIGURE S1.10**

Trend in allele frequency of loci that become fixed in the 50 generations of selection for the five selection methods and three genetic models. For all loci, the favorable allele was counted, therefore red lines indicate loci fixed for the favorable allele and yellow lines indicate loci fixed for the unfavorable allele, based on the statistical additive effect in generation 0. The minimum change in allele frequency was set at 0.2. The five selection methods were: RANDOM selection, MASS selection, PBLUP selection with own performance (PBLUP_OP), GBLUP selection without own performance (GBLUP_NoOP) or with own performance (GBLUP_OP). The three genetic models were a model with only additive effects (A), with additive and dominance effects (AD), or with additive, dominance and epistatic effects (ADE). Results are shown for one replicate.


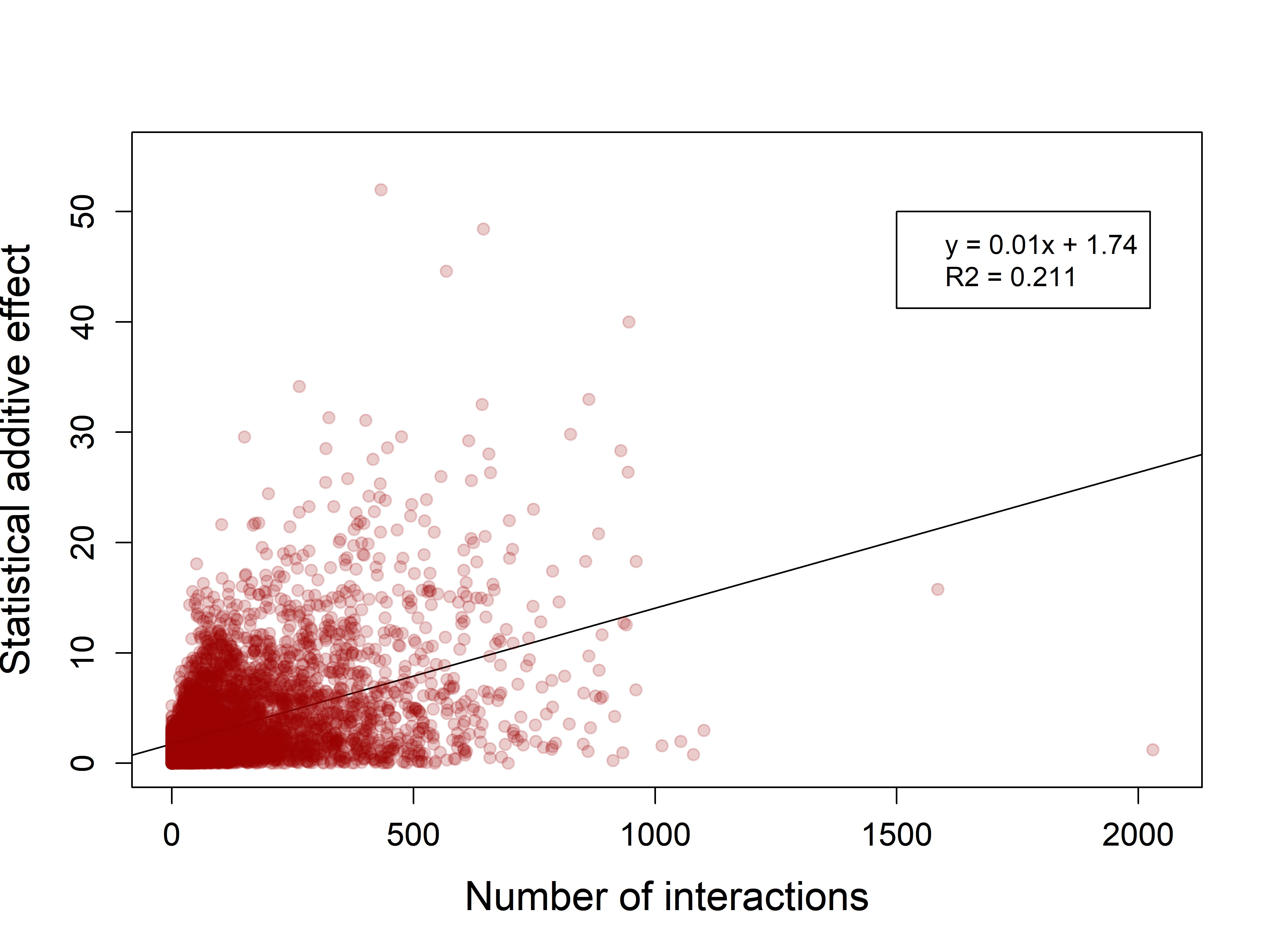


**FIGURE S1.11**

Correlation between the number of interactions at a causal locus and its absolute statistical additive effect for the genetic model with additive, dominance and epistatic effects (ADE).


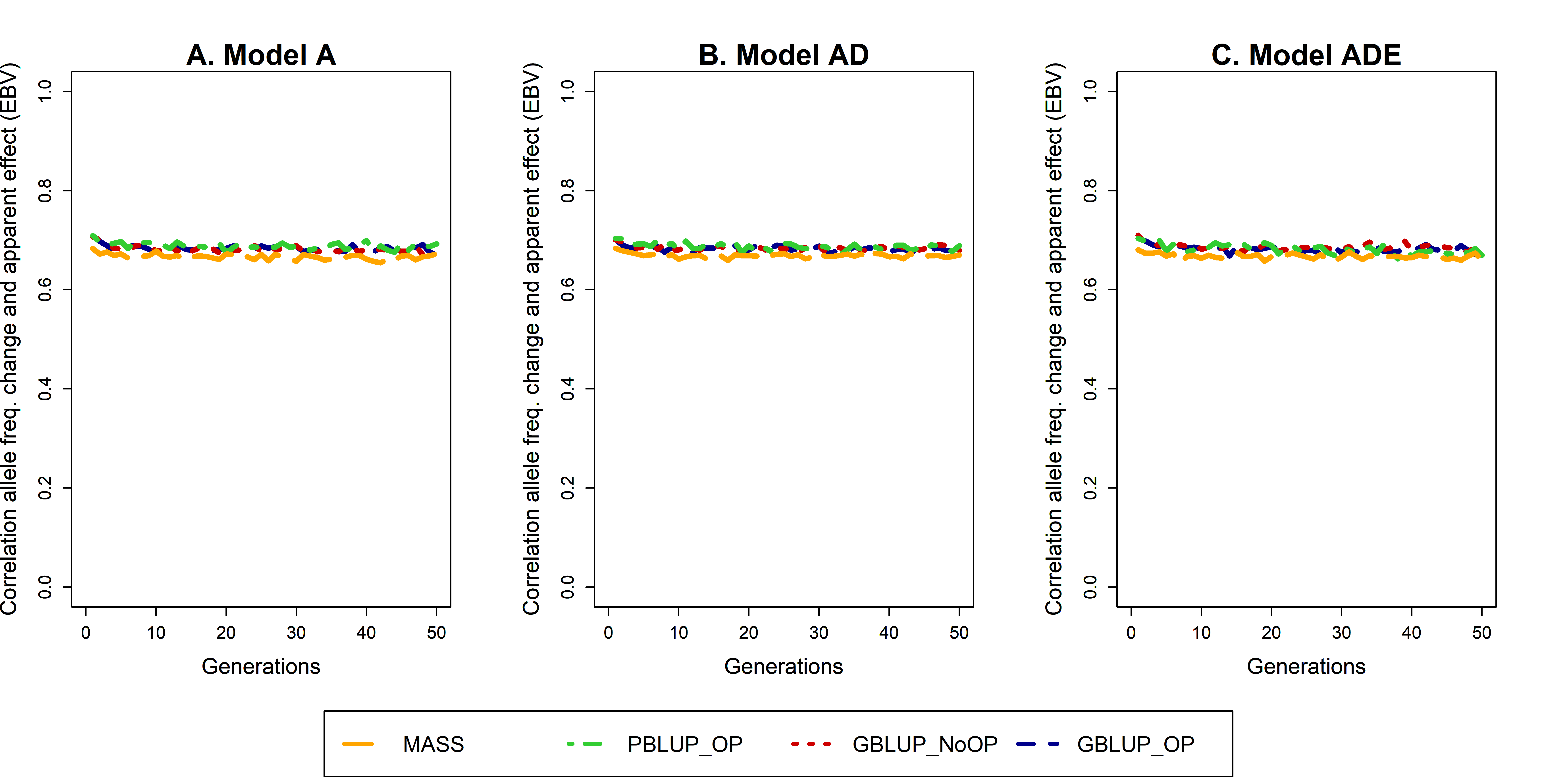


**FIGURE S1.12**

Correlation between the change in allele frequency towards the next generation and the apparent effect of a locus based on the estimated breeding values for four selection methods and three genetic models. The change in allele frequency is expressed as the absolute change in allele frequency from generation *i* to generation *i* +1 divided by *p_i_*(1-*p_i_*), where *p_i_* is the allele frequency in generation *i*. The four selection methods were: MASS selection, PBLUP selection with own performance (PBLUP_OP), GBLUP selection without own performance (GBLUP_NoOP) or with own performance (GBLUP_OP). The three genetic models were a model with only additive effects (A), with additive and dominance effects (AD), or with additive, dominance and epistatic effects (ADE). Results are shown as averages of 20 replicates.


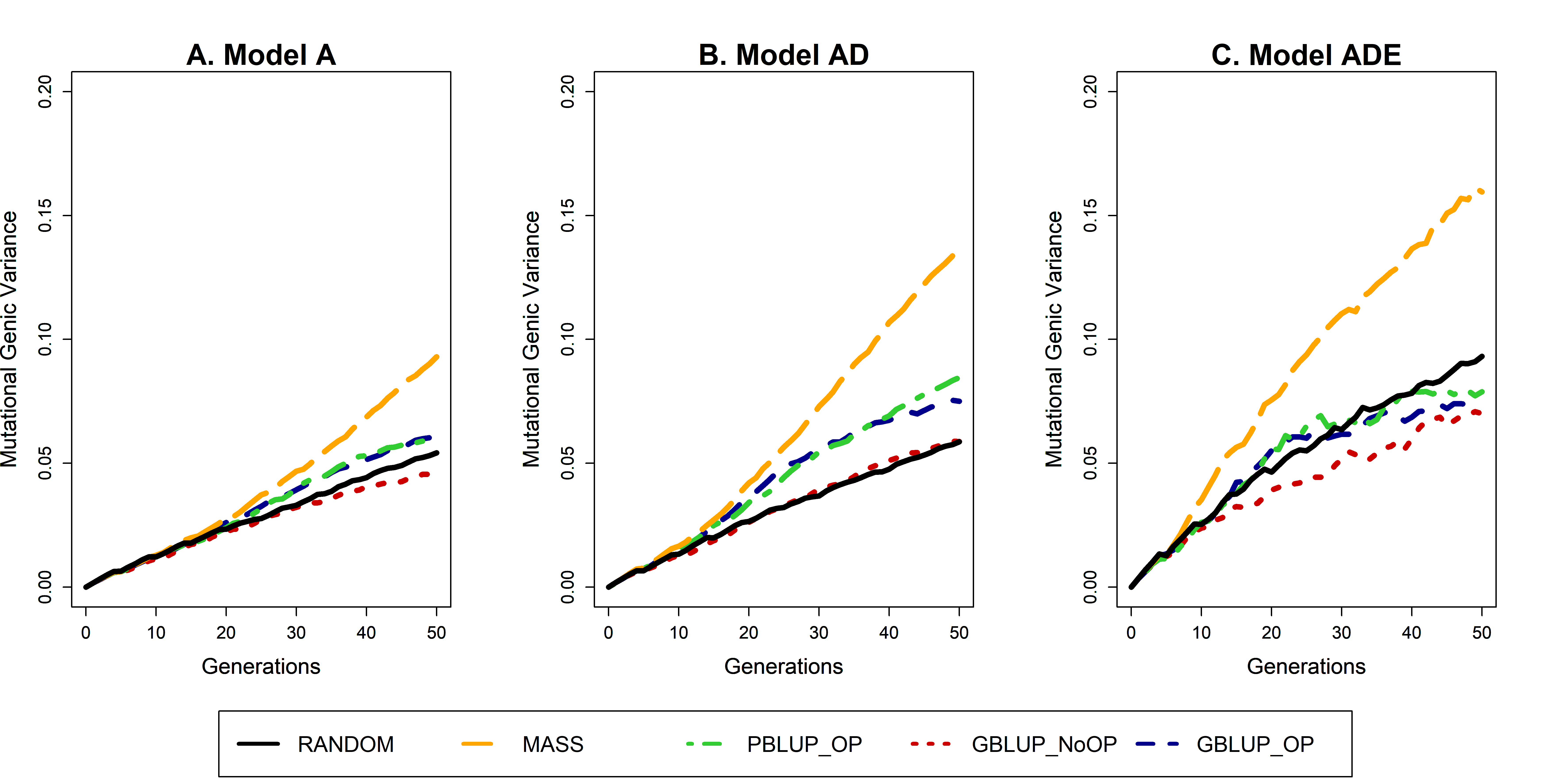


**FIGURE S1.13**

Mutational genic variance for the five selection methods and three genetic models. The mutational genic variance was calculated in each generation as the sum of *2p_i_(1-p_i_)*$\alpha_{i}^{2}$ across all segregating mutations *i* with allele frequency *p* and statistical additive effect *α*. For comparison across genetic models, the mutational genic variance was scaled by the statistical additive genetic variance in generation 0. The five selection methods were: RANDOM selection, MASS selection, PBLUP selection with own performance (PBLUP_OP), GBLUP selection without own performance (GBLUP_NoOP) or with own performance (GBLUP_OP). The three genetic models were a model with only additive effects (A), with additive and dominance effects (AD), or with additive, dominance and epistatic effects (ADE). Results are shown as averages of 20 replicates.
