# Supplementary material for: The long-term effects of genomic selection: 2. Changes in allele frequencies of causal loci and new mutations
Source: Genetics. 2023 Jul 28;225(1):iyad141. doi: 10.1093/genetics/iyad141 (PMC10471209; doi:10.1093/genetics/iyad141)
Supplement: iyad141_Supplementary_Data [file iyad141_supplementary_data.zip › File_S2_GENETICS-2023-306366.docx]

**File S2: Supplementary tables**

**Table S2.1 –** Total number of mutations, number of mutations lost after the first generation, and proportion of mutations lost after the first generation for the five selection methods and three genetic models^1^. The five selection methods were: RANDOM selection, MASS selection, PBLUP selection with own performance (PBLUP_OP), GBLUP selection without own performance (GBLUP_NoOP) or with own performance (GBLUP_OP). The three genetic models were a model with only additive effects (A), with additive and dominance effects (AD), or with additive, dominance and epistatic effects (ADE).

|  | **Total number of mutations** | | **Number of mutations lost in 1 generation** | | **Proportion lost** | |
| --- | --- | --- | --- | --- | --- | --- |
| ***Model A*** |  |  |  |  |  |  |
| **RANDOM** | 31,180 | (7.2) | 24,979 | (18.9) | 0.80 | (0.001) |
| **MASS** | 31,284 | (6.4) | 25,039 | (19.7) | 0.80 | (0.001) |
| **PBLUP_OP** | 31,556 | (5.7) | 25,254 | (24.0) | 0.80 | (0.001) |
| **GBLUP_NoOP** | 31,429 | (5.8) | 25,183 | (17.6) | 0.80 | (0.001) |
| **GBLUP_OP** | 31,416 | (5.4) | 25,126 | (14.7) | 0.80 | (0.000) |
|  |  |  |  |  |  |  |
| ***Model AD*** |  |  |  |  |  |  |
| **RANDOM** | 31,176 | (8.1) | 24,940 | (14.6) | 0.80 | (0.000) |
| **MASS** | 31,264 | (7.5) | 24,969 | (23.2) | 0.80 | (0.001) |
| **PBLUP_OP** | 31,546 | (4.3) | 25,217 | (17.5) | 0.80 | (0.001) |
| **GBLUP_NoOP** | 31,430 | (4.7) | 25,133 | (20.6) | 0.80 | (0.001) |
| **GBLUP_OP** | 31,411 | (5.2) | 25,140 | (19.1) | 0.80 | (0.001) |
|  |  |  |  |  |  |  |
| ***Model ADE*** |  |  |  |  |  |  |
| **RANDOM** | 31,188 | (7.1) | 24,972 | (17.1) | 0.80 | (0.000) |
| **MASS** | 31,281 | (6.3) | 25,060 | (16.3) | 0.80 | (0.000) |
| **PBLUP_OP** | 31,583 | (2.5) | 25,296 | (15.7) | 0.80 | (0.000) |
| **GBLUP_NoOP** | 31,466 | (4.8) | 25,186 | (17.6) | 0.80 | (0.001) |
| **GBLUP_OP** | 31,453 | (4.9) | 25,184 | (16.9) | 0.80 | (0.000) |

^1^ Results are shown as averages across replicates with their corresponding standard errors of the mean between brackets.

**Table S2.2 –** Correlation and regression coefficient of the regression of the change in allele frequency over 50 generations on the statistical additive effect scaled by the statistical additive genetic standard deviation in generation 0 for the five selection methods and three genetic models. Only loci that were segregating in generation 0 were considered. The five selection methods were: RANDOM selection, MASS selection, PBLUP selection with own performance (PBLUP_OP), GBLUP selection without own performance (GBLUP_NoOP) or with own performance (GBLUP_OP). The three genetic models were a model with only additive effects (A), with additive and dominance effects (AD), or with additive, dominance and epistatic effects (ADE). Results are given for one replicate.

|  | **Model A** | **Model AD** | **Model ADE** |
| --- | --- | --- | --- |
| ***Correlation coefficient*** |  |  |  |
| **RANDOM** | -0.018 | -0.027 | -0.001 |
| **MASS** | 0.410 | 0.421 | 0.327 |
| **PBLUP_OP** | 0.331 | 0.334 | 0.243 |
| **GBLUP_NoOP** | 0.327 | 0.369 | 0.247 |
| **GBLUP_OP** | 0.385 | 0.424 | 0.275 |
|  |  |  |  |
| ***Regression coefficient*** |  |  |  |
| **RANDOM** | -0.032 | -0.043 | -0.001 |
| **MASS** | 1.754 | 1.619 | 0.872 |
| **PBLUP_OP** | 1.708 | 1.457 | 0.803 |
| **GBLUP_NoOP** | 1.813 | 1.801 | 0.940 |
| **GBLUP_OP** | 2.220 | 2.166 | 1.053 |

**Table S2.3 –** Percentage of loci that changed bin number compared to generation 0 for the five selection methods and three genetic models. The bin number was assigned in each generation based on the size of the statistical additive effect. The five selection methods were: RANDOM selection, MASS selection, PBLUP selection with own performance (PBLUP_OP), GBLUP selection without own performance (GBLUP_NoOP) or with own performance (GBLUP_OP). The three genetic models were a model with only additive effects (A), with additive and dominance effects (AD), or with additive, dominance and epistatic effects (ADE). With only additive or additive and dominance effects, the following ranges in values of statistical additive effects were used: bin 1: < 0.2, bin 2: 0.2 – 0.4, bin 3: 0.4 – 0.6, bin 4: 0.6 – 0.8, bin 5: 0.8 – 1.0, bin 6: 1.0 – 1.2, bin 7: 1.2 – 1.4, bin 8: 1.4 – 1.6, bin 9: 1.6 – 1.8, bin 10: 1.8 – 2.0, bin 11: 2.0 – 2.2, bin 12: 2.2 – 2.4, bin 13: 2.4 – 2.6, bin 14: 2.6 – 2.8, bin 15: >2.8. With additive, dominance and epistasis effects, the following ranges in values of statistical additive effects were used: bin 1: < 0.5, bin 2: 0.5 – 1.0, bin 3: 1.0 – 2.0, bin 4: 2.0 – 3.0, bin 5: 3.0 – 4.0, bin 6: 4.0 – 5.0, bin 7: 5.0 – 6.0, bin 8: 6.0 – 7.0, bin 9: 7.0 – 8.0, bin 10: 8.0 – 10.0, bin 11: 10.0 – 12.0, bin 12: 12.0 – 14.0, bin 13: 14.0 – 16.0, bin 14: 16.0 – 20.0, bin 15: >20.0.

|  | **Percentage of loci changing bin compared to generation 0** | | | | |
| --- | --- | --- | --- | --- | --- |
|  | **Gen. 10** | **Gen. 20** | **Gen. 30** | **Gen. 40** | **Gen. 50** |
| ***Model A*** |  |  |  |  |  |
| **RANDOM** | 0.0 | 0.0 | 0.0 | 0.0 | 0.0 |
| **MASS** | 0.0 | 0.0 | 0.0 | 0.0 | 0.0 |
| **PBLUP_OP** | 0.0 | 0.0 | 0.0 | 0.0 | 0.0 |
| **GBLUP_NoOP** | 0.0 | 0.0 | 0.0 | 0.0 | 0.0 |
| **GBLUP_OP** | 0.0 | 0.0 | 0.0 | 0.0 | 0.0 |
|  |  |  |  |  |  |
| ***Model AD*** |  |  |  |  |  |
| **RANDOM** | 6.5 | 7.8 | 8.8 | 9.5 | 10.1 |
| **MASS** | 9.6 | 12.6 | 14.7 | 16.3 | 17.4 |
| **PBLUP_OP** | 10.8 | 14.0 | 15.9 | 17.2 | 18.0 |
| **GBLUP_NoOP** | 11.3 | 14.5 | 16.3 | 17.3 | 18.1 |
| **GBLUP_OP** | 11.2 | 14.5 | 16.5 | 17.6 | 18.4 |
|  |  |  |  |  |  |
| ***Model ADE*** |  |  |  |  |  |
| **RANDOM** | 24.7 | 29.7 | 32.9 | 35.7 | 37.9 |
| **MASS** | 35.1 | 44.5 | 49.4 | 52.7 | 55.1 |
| **PBLUP_OP** | 41.6 | 49.2 | 53.7 | 55.8 | 57.4 |
| **GBLUP_NoOP** | 42.3 | 49.8 | 53.7 | 55.7 | 57.1 |
| **GBLUP_OP** | 43.0 | 50.6 | 54.7 | 56.9 | 58.2 |

**TABLE S2.4** – Average number of mutations per replicate that became fixed for the newly derived allele over 50 generations of selection for the five selection methods and three genetic models^1^. The five selection methods were: RANDOM selection, MASS selection, PBLUP selection with own performance (PBLUP_OP), GBLUP selection without own performance (GBLUP_NoOP) or with own performance (GBLUP_OP). The three genetic models were a model with only additive effects (A), with additive and dominance effects (AD), or with additive, dominance and epistatic effects (ADE).

|  | **Model A** | **Model AD** | **Model ADE** |
| --- | --- | --- | --- |
| **RANDOM** | 0.00 | 0.00 | 0.00 |
| **MASS** | 0.00 | 0.00 | 0.00 |
| **PBLUP_OP** | 0.45 | 0.25 | 0.05 |
| **GBLUP_NoOP** | 0.65 | 0.15 | 0.05 |
| **GBLUP_OP** | 0.65 | 0.25 | 0.15 |

^1^ Results are shown as averages across replicates.
