# Supplementary material for: The long-term effects of genomic selection: 2. Changes in allele frequencies of causal loci and new mutations
Source: Genetics. 2023 Jul 28;225(1):iyad141. doi: 10.1093/genetics/iyad141 (PMC10471209; doi:10.1093/genetics/iyad141)
Supplement: iyad141_Supplementary_Data [file iyad141_supplementary_data.zip › File_S3_GENETICS-2023-306366.docx]

**File S3: Adjusting the number of parents to change the effective population size (*N­_e_*) to the desired value**

Under random selection and when matings are monogamous (i.e., when the number of selected males (*N_m_*) equals the number of selected females (*N_f_*)), the effective population size can be estimated as (Falconer and Mackay 1996):

$N_{e}=\frac{4\left( N_{m}+N_{f} \right)}{V_{k}+2},$ (S4.1)

where *V_k_* represents the variance of family size.

In our simulations, family size before selection is fixed, therefore, the variation in family size after selection follows a hypergeometric distribution:

$V_{k}=n\frac{K}{N} \frac{\left( N-K \right)}{N} \frac{\left( N-n \right)}{\left( N-1 \right)},$ (S4.2)

where *N* is the number of selection candidates, *n* is the number of selected individuals (*n*=$N_{m}+N_{f}$), and *K* is the family size before selection.

In our random selection scenario, *N*=1000, *n*=200, and K=10. Therefore, *V_k_* = 1.585, and *N_e_*=1.116*n*=223.

For the other scenarios, we set the estimated *N_e_* based on pedigree inbreeding coefficient as the desired *N_e_*. We used this *N_e_* to estimate *n* that would result in the same *N_e_* under random selection as *n=N_e_*/1.116 and set *N_m_*=*N_f_*=0.5**n*. The family size before selection was set at 10. In the table below, the estimated *n*, *N_m_*, *N_f_*, *K*, *N* are shown, as well as the resulting *V_k_* (using equation S4.2), the estimated *N_e_* (using equation S4.1) and the simulated *N_e_* using those parameters under random selection based on the pedigree inbreeding coefficient.

**Table S3.1** – Estimation of the required number of selected parents to end up at the desired effective population size under random selection for the different selection scenarios.

|  | **Desired *N_e_*** | ***n*** | ***N*_m_=*N_f_*** | ***K*** | ***N*** | ***V_k_*** | **Estimated *N_e_*** | **Simulated *N_e_*** |
| --- | --- | --- | --- | --- | --- | --- | --- | --- |
| **MASS** | 143 | 128 | 64 | 10 | 640 | 1.577 | 143 | 143 |
| **PBLUP_OP** | 47 | 42 | 21 | 10 | 210 | 1.531 | 48 | 47 |
| **GBLUP_NoOP** | 72 | 64 | 32 | 10 | 320 | 1.555 | 72 | 72 |
| **GBLUP_OP** | 81 | 72 | 36 | 10 | 360 | 1.560 | 81 | 80 |

# Literature cited

Falconer DS, Mackay TFC. 1996. Introduction to quantitative genetics. Harlow, United Kingdom: Pearson Education Limited.
